# Kartogenin-loaded chitosan composite scaffold with cartilage-mimetic microstructure for layered osteochondral repair and cartilage phenotype maintenance

**DOI:** 10.1016/j.mtbio.2025.102727

**Published:** 2025-12-22

**Authors:** Hengyu Liu, Hongqing Qiao, Rudong Li, Wenbo Yang, Xingchen Guo, Yuhang Wang, Nan Mei, Jincheng Wang, Fei Chang

**Affiliations:** a Department of Orthopedic Surgery, The Second Hospital of Jilin University, Changchun 130041, PR China; b Department of Gastrointestinal Nutrition and Hernia Surgery, The Second Hospital of Jilin University, Changchun 130041, PR China; c Department of Orthopaedic Surgery, Nara Medical University, Kashihara 634-8521, Japan; d Health Technology College, Jilin Sport University, Changchun 130022, PR China

**Keywords:** 3D printing, Chitosan, Cartilage microstructure, Layered repair, Gradient structure, Osteochondral

## Abstract

Osteochondral defects represent a formidable clinical challenge due to their distinctive stratified architecture and limited intrinsic healing capacity. However, conventional tissue engineering scaffolds exhibit significant limitations in replicating the gradient properties of native osteochondral tissue while maintaining chondrogenic phenotypes. This study presents a novel bio-customized gradient scaffold CSK@G inspired by natural osteochondral architecture, developed through strategic material composition control and graduated porosity design techniques, for integrated repair of osteochondral defects. Through precise biomimetic design, the scaffold successfully recapitulates the spatial heterogeneity of native tissue by accurately mimicking five anatomically distinct layers—superficial cartilage, middle cartilage, deep cartilage, calcified cartilage, and subchondral bone. Additionally, Kartogenin (KGN)-loaded Chitosan (CS) hydrogels are strategically incorporated within cartilaginous zones to facilitate stem cell homing and create optimal cellular microenvironments that provide region-specific biochemical cues for enhanced chondrogenic differentiation. Comprehensive experimental results, both *in vitro* and *in vivo*, demonstrate that this bio-customized gradient scaffold significantly facilitates integrated osteochondral repair while effectively maintaining chondrogenic phenotypes. This work proposes a novel bio-customized strategy for osteochondral tissue engineering, resulting in a bio-customized scaffold that closely mimics the hierarchical properties of natural osteochondral tissue.

## Highlights

• Mimics the five-layer microstructure of native osteochondral tissue, reproducing its gradient spatial heterogeneity.
• Integrates KGN-loaded hydrogel in cartilage zone to direct stem cell homing and chondrogenic differentiation.
• Employs gradient porosity to preserve chondrogenic phenotype and prevent dedifferentiation.
• Utilizes accessible materials and scalable fabrication for reproducible clinical translation.

## Graphical abstract

**Graphical abstract** This study presents a comprehensive approach for osteochondral repair through gradient scaffold construction. The process begins with 3D printing technology to fabricate gradient scaffolds, followed by incorporating CS as the primary component mixed with and KGN to fill the chondral layer pores. After composite scaffold assembly, the therapeutic efficacy was evaluated through both ectopic experiments and orthotopic experiments.

**Figure:**
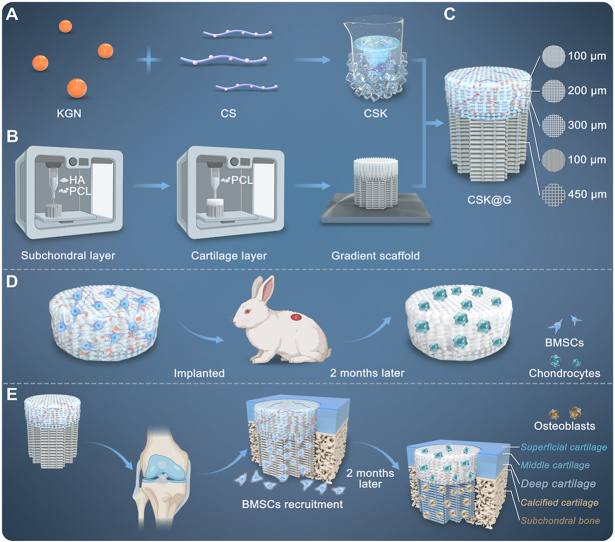

## 1 Introduction

Under normal physiological conditions, chondrocytes maintain continuous bidirectional interactions with neighboring osteoblasts and osteoclasts within the subchondral bone [[1], [2], [3]]. Given this interdependent relationship, cartilage and subchondral bone should be conceptualized as an integrated osteochondral unit [4]. Osteochondral defects represent an increasingly prominent clinical challenge, causing pain, loss of function, and disability [5]. Although treatments including Autologous Chondrocyte Implantation (ACI), Matrix-induced Autologous Chondrocyte Implantation (MACI), and microfracture have been widely adopted in clinical practice, they still face significant limitations such as limited tissue availability, poor integration, and progressive ossification of regenerated cartilage [6,7]. Therefore, achieving optimal cartilage regeneration and maintaining cartilage phenotype and function remains a critical challenge in regenerative medicine [8].

Natural osteochondral tissue exhibits five continuous but distinct zones in the vertical direction: superficial cartilage, middle cartilage, deep cartilage, calcified cartilage, and subchondral bone layers. The superficial cartilage layer, located at the outermost region, possesses a dense structure and intact natural barrier that prevents the infiltration of macromolecular proteins from synovial fluid [9]. The middle and deep cartilage zones demonstrate significant compressive resistance [10]. The calcified cartilage layer, situated at the base of the cartilage, serves as a transitional zone between non-calcified cartilage and mineralized bone [11,12]. The subchondral bone layer, located at the bottommost region, provides mechanical support and participates in nutrient exchange with overlying cartilage. Currently, tissue-engineered osteochondral scaffolds have emerged as a promising strategy for simultaneous regeneration of cartilage and subchondral bone [13].

Recent advances in biomaterial design have increasingly emphasized the importance of gradient architectures and functional optimization in osteochondral tissue engineering. Notably, dual-gradient silk-based hydrogel systems that integrate both structural and biochemical gradients have demonstrated superior capacity in spatially targeted delivery and zone-specific tissue regeneration [14]. Such gradient designs enable precise control over cellular microenvironments, facilitating the formation of distinct cartilage and bone layers. Additionally, thermo-sensitive poly (amino acid) hydrogels have emerged as promising carriers that mediate cytoprotection through antioxidant mechanisms, offering advantages in injectability and in situ gelation at physiological temperature [15]. Furthermore, innovative scaffold fabrication strategies, such as thinning electrospun scaffolds to achieve tissue thickening, have been developed to enhance cell infiltration and tissue integration [16]. Biomaterial approaches incorporating bioactive components for dentin desensitization have also provided insights into designing scaffolds with enhanced bioactivity and cellular responsiveness [17]. These studies collectively highlight the potential of biomimetic gradient scaffolds in recapitulating the complex osteochondral interface.

However, most conventional scaffolds fail to recapitulate the natural multi-layered osteochondral structure, leading to suboptimal repair outcomes or inability to maintain cartilage phenotype and function, resulting in progressive ossification and compromised long-term therapeutic efficacy [18]. The design of novel scaffolds should thoroughly mimic the intricate structural characteristics and functional zonation of natural osteochondral tissue to achieve authentic biomimicry and functional reconstruction. Therefore, from a bio-customized perspective, ideal scaffolds should not only facilitate cartilage repair but also maintain the phenotype and function of newly formed cartilage while possessing spatial gradient characteristics to enable stratified induction and simulate the structural and functional features of natural osteochondral tissue. Consequently, the fabrication of scaffolds that conform to the natural osteochondral gradient structure represents a critical approach for achieving both effective cartilage repair and sustained maintenance of cartilage phenotype and function.

In terms of material selection, polycaprolactone (PCL) has been extensively utilized in cartilage scaffold fabrication due to its excellent biocompatibility, appropriate elastic modulus, and FDA approval [19]. Hydroxyapatite (HA), as the primary inorganic component of bone tissue, represents an ideal bone repair material that significantly promotes osteogenic differentiation of stem cell [20]. The incorporation of HA into PCL not only enhances mechanical properties and osteogenic capacity but also facilitates calcified cartilage formation. To enhance bioactivity, we incorporated Kartogenin (KGN)-loaded Chitosan (CS) hydrogel onto the cartilaginous layer [21]. KGN specifically promotes BMSC chondrogenic differentiation by activating CBF-β/RUNX1 signaling, thereby enhancing cartilage ECM deposition and accelerating tissue regeneration. As a natural polysaccharide with excellent biocompatibility and biodegradability, CS after crosslinking enables sustained KGN release and undergoes thermosensitive gelation at physiological temperature (37 °C), creating an optimal microenvironment for chondrogenesis and facilitating clinical translation [22].

Therefore, inspired by the natural osteochondral structure, particularly the differences in extracellular matrix density and cell morphology across different osteochondral layers, this study aims to design a bio-customized gradient osteochondral scaffold CSK@G that replicates this spatial heterogeneity by precisely controlling the pore size gradient, ranging from dense pores in the cartilage layer to larger pores in the bone layer. This gradient architecture not only facilitates osteochondral regeneration but also maintains cartilage phenotype and function to achieve layered regeneration of osteochondral tissue. Using PCL as the main material, combined with HA and KGN-loaded CS hydrogel, we designed and constructed a bio-customized gradient scaffold that recapitulates the multi-layered architecture of natural osteochondral tissue. The CS-KGN system was specifically incorporated into the cartilage layers to enhance chondrogenic differentiation and phenotype maintenance. Subsequently, we systematically evaluated the physicochemical properties of this bio-customized gradient scaffold and assessed its biocompatibility and regulatory effects on osteogenic and chondrogenic differentiation through *in vitro* experiments. Through *in vivo* ectopic and orthotopic experiments, we comprehensively investigated the potential application of this scaffold in osteochondral defect repair and validated its capacity to maintain cartilage phenotype.

## 2 Materials and methods

### 2.1 Fabrication of 3D-printed porous scaffolds

The materials included commercially available PCL (molecular weight = 80,000, RHAWN, Shanghai, China) and HA powder (Aladdin, Shanghai, China). The PCL/HA composite material used for scaffold fabrication was synthesized by mixing 20 % (w/w) HA powder with molten PCL at 120 °C [23]. We designed cylindrical scaffolds with gradient pore sizes (5 mm in diameter × 5 mm in height) for animal experiments to evaluate *in vivo* osteochondral repair. The STL files of these models were imported into SolidWorks software (SolidWorks Corp., Concord, MA, USA) for design and digitally sliced using Simplify3D software (Simplify3D LLC, Cincinnati, OH, USA) to generate 2D construction files. The gradient scaffold was designed with a height of 5 mm, divided into 25 layers with a layer thickness of 200 μm each. The scaffold consisted of five distinct sections: PCL with 100 μm pores, 2 layers (superficial cartilage); PCL with 200 μm pores, 3 layers (middle cartilage); PCL with 300 μm pores, 3 layers (deep cartilage); PCL/HA with 100 μm pores, 2 layers (calcified cartilage); and PCL/HA with 450 μm pores, 15 layers (subchondral bone). This gradient scaffold was designated as “G". Additionally, we fabricated a control scaffold with the same materials but uniform 450 μm pores throughout: PCL, 8 layers; PCL/HA, 17 layers, designated as “S".

Additionally, we fabricated four uniform pore-size cylindrical scaffolds (5 mm diameter × 2 mm height) corresponding to specific cartilage layers: P100 (PCL, 100 μm pores) for superficial cartilage, P200 (PCL, 200 μm pores) for middle cartilage, P300 (PCL, 300 μm pores) for deep cartilage, and P/H100 (PCL/HA, 100 μm pores) for calcified cartilage. These reduced-height scaffolds enabled separate *in vitro* evaluation of individual layers to verify layer-specific expression patterns. During 3D printing, the FDM printer was heated to 110 °C for PCL and PCL/HA processing. Semi-solid filaments were extruded at 90° angles by moving the substrate along X and Y axes. Scaffolds were manufactured layer-by-layer, with gradient scaffolds printed by fabricating PCL/HA layers first, then switching to PCL layers. Pore sizes were controlled by adjusting infill rate and printing speed. Printed scaffolds were sterilized by UV irradiation for 24 h.

### 2.2 Preparation of KGN-loaded CS and polymer composite scaffolds

First, 0.5 mL of glacial acetic acid (Sigma-Aldrich, Beijing, China) was dissolved in 50 mL of deionized water to prepare a 1 % acetic acid solution. Then, 1 g of CS powder (Cat. No. 419419, Mw: 310–375 kDa, degree of deacetylation ≥75 %, viscosity: 800–2000 cP in 1 % acetic acid at 25 °C; Sigma-Aldrich, Beijing, China) was added to this solution and vigorously stirred for 2 h to prepare a 2 % CS solution, followed by debubbling at 4 °C. Subsequently, 5.6 g of β-glycerophosphate disodium salt (β-GP, Sigma-Aldrich, Shanghai, China) was dissolved in 7.5 mL of deionized water to prepare a 56 % β-GP solution. Based on our preliminary dose-screening experiments (0, 1, 5, 10 μM) [24], a KGN concentration of 5 μM was selected for this experiment due to its optimal chondrogenic differentiation efficacy.

Therefore, 20 μg of KGN (Sigma-Aldrich, Shanghai, China) was dissolved in 4 mL of DMSO (Sigma-Aldrich, Shanghai, China) to obtain a 5 μM KGN solution. To conjugate KGN with the CS hydrogel, 50 μL of 1-ethyl-3-(3-dimethylaminopropyl)carbodiimide (EDC, 0.5 M, Sigma-Aldrich, Shanghai, China) and 100 μL of N-hydroxysuccinimide (NHS, 0.1 M, Sigma-Aldrich, Shanghai, China) cross-linking solution were first added to activate the amino groups (-NH_2_) in the CS solution. Next, the KGN solution was mixed with the CS solution and allowed to react at room temperature to form stable amide bonds (-CONH-), achieving covalent bonding between KGN and CS. Subsequently, 0.375 mL of KGN solution was added to 7.5 mL of β-GP solution, and 22.5 mL of CS solution was added dropwise under ice bath conditions, followed by vigorous stirring and reaction in a 37 °C water bath for 30 min to complete the gelation process. During sterilization, the CS solution was autoclaved at 121 °C for 20 min, while the β-GP and KGN solutions were filtered through 0.22 μm filters. The entire synthesis process was conducted in a clean operating cabinet.

A vacuum pump was employed to infiltrate the hydrogel into the microporous architecture of the 3D-printed scaffold cartilage layers, yielding composite scaffolds for systematic experimental evaluation. The cartilage layers were stratified into distinct experimental groups according to scaffold material composition and pore size specifications. The KGN-CS hydrogel (designated CSK) was selectively infiltrated into various scaffold configurations: CSK@P100 (CSK infiltrated into PCL scaffolds with 100 μm pores), CSK@P200 (200 μm pore PCL scaffolds), CSK@P300 (300 μm pore PCL scaffolds), and CSK@P/H100 (PCL/HA composite scaffolds with 100 μm pores). These composite scaffolds were subsequently employed for subcutaneous implantation experiments. The detailed grouping design for subcutaneous implantation is presented in Table 2. Furthermore, for the articular cartilage defect model, CSK was infiltrated into the cartilage regions of the gradient pore-size scaffold G (CSK@G) and the uniform pore-size scaffold S (CSK@S), establishing a comprehensive experimental matrix for comparative analysis. The detailed grouping design for the articular experiment is presented in Table 3.

**Table 1:** Experimental group setup for mechanical testing.

Group name	Cartilage layer	Bone layer	Gradient transition
PCL	PCL	PCL	–
PCL/HA	PCL/HA	PCL/HA	–
S	PCL	PCL/HA	–
G	PCL	PCL/HA	+

**Table 2:** Experimental group setup for subcutaneous implantation.

Group name	Pore size	HA	Mimicked cartilage zone
CSK@P100	100 μm	–	Superficial zone
CSK@P200	200 μm	–	Middle zone
CSK@P300	300 μm	–	Deep zone
CSK@P/H100	100 μm	+	Calcified cartilage zone

**Table 3:** Experimental group setup for the articular cartilage defect model.

Group name	CSK in cartilage layer	Gradient transition
S	–	–
G	–	+
CSK@S	+	–
CSK@G	+	+

### 2.3 Material characterization of scaffolds and CS hydrogels

#### 2.3.1 Morphological observation

The internal microstructure and surface of the fiber networks were observed using scanning electron microscopy (SEM; SU-8100, Hitachi, Japan). ImageJ software (National Institutes of Health, Bethesda, Maryland, USA) was used for analysis.

#### 2.3.2 Phase and structural analysis

The phase and structure of the materials were characterized using Fourier Transform Infrared Spectroscopy (FTIR; Thermo Fisher Scientific Inc., Madison, WI, USA). FTIR was used to observe the characteristics of specific materials in the wavenumber range of 500–4000 cm^−1^.

#### 2.3.3 Compressive strength testing of scaffolds

The uniaxial unconfined compression tests were conducted using a hydraulic testing machine (MTS MiniBionix, Minneapolis, USA) equipped with a 10 N load cell to evaluate the compressive mechanical properties of different scaffold types, including scaffolds made entirely of cartilage layer material (PCL), scaffolds made entirely of bone layer material (PCL/HA), conventional bilayer scaffolds (S), and graded bilayer scaffolds (G) (Table 1, n = 3). All samples were uniform cylinders (5 mm in diameter and 5 mm in height), and compression was applied at a rate of 1 mm/min. The compressive Young's modulus was determined by calculating the slope of the stress-strain curve within the 10 %–15 % strain range.

#### 2.3.4 Compressive modulus testing of scaffolds

Using the same hydraulic testing machine and 10 N load cell, uniaxial unconfined compression tests were performed on scaffolds (pure PCL, pure PCL/HA, S, and G) (n = 3). Test conditions were identical to those of the compressive strength testing, with all samples being uniform cylinders (5 mm in diameter and 5 mm in height) and loaded at a rate of 1 mm/min. The compressive modulus was determined by calculating the slope of the stress-strain curve within the 5 %–10 % strain range to evaluate the scaffolds' resistance to deformation.

#### 2.3.5 Shear performance testing of scaffolds

The shear mechanical properties of different scaffolds (pure PCL, pure PCL/HA, S, and G) (n = 3) were evaluated using a universal testing machine (MTS, Instron Electropuls E10000, USA). Samples were placed in specialized fixtures to ensure scaffold stability during the shear process. Lateral shear force was applied at a rate of 1 mm/min during the shear test, and stress-strain curves were recorded. The shear modulus was calculated from the slope of the stress-strain curve within the 0.5–1 mm displacement range to analyze the shear deformation performance of the scaffolds.

#### 2.3.6 Water contact angle

The hydrophilicity was tested using single-layer thin film samples (5 mm × 5 mm) of different materials, and the contact angles were calculated using CA100D static/dynamic contact angle testing software.

### 2.4 In vitro degradation rate of CS hydrogels

To evaluate the *in vitro* degradation rate of hydrogels, freeze-dried hydrogel samples were initially prepared by freezing at −80 °C, followed by lyophilization and weighing to obtain the initial dry weight (W_0_). The dried hydrogel samples were then immersed in 3 mL of lysozyme solution (10 mg/mL) and incubated in a cell culture incubator at 37 °C. Samples were retrieved at specific time points (days 0, 1, 2, 3, 4, 5, 6, 7, 8, 10, 12, and 14) with three samples per time point (n = 3). Retrieved samples were gently rinsed with distilled water, freeze-dried again, and weighed to obtain the remaining dry weight (W_t_). The degradation rate was calculated as: Degradation rate (%) = (W_0_ − W_t_)/W_0_ × 100. The remaining weight ratio was calculated as: Remaining weight ratio (%) = W_t_/W_0_ × 100.

### 2.5 In vitro release of KGN from composite scaffolds

KGN solutions of varying concentrations were prepared in PBS, and analyzed using High-Performance Liquid Chromatography (HPLC) with UV detection at 270 nm to construct a calibration curve. To evaluate the release rate of KGN from composite scaffolds, CS hydrogels loaded with 5 μM KGN were immersed in PBS containing lysozyme (10 mg/mL) to simulate the enzymatic degradation environment *in vivo* and incubated in a water bath (37 °C, 60 rpm). At specific time points (days 0, 1, 2, 3, 4, 5, 6, 7, 8, 9, 10, 11, 12, 13, 14, and 15), 1 mL release medium samples were collected and immediately replenished with an equal volume of fresh lysozyme-containing PBS. The concentration of released KGN in the samples was analyzed using the established HPLC method, and a cumulative release curve was plotted.

### 2.6 In vitro cell experiments

#### 2.6.1 rBMSCs culture

Rabbit bone marrow mesenchymal stem cells (rBMSCs, Pricella, China) were cultured in complete medium consisting of F-12 basal medium (Sigma, USA), 10 % fetal bovine serum (Gibco, USA), and 1 % penicillin/streptomycin (Hyclone, USA). Cells were maintained in an incubator at 37 °C with 5 % CO2. The culture medium was changed every 3 days.

#### 2.6.2 Cell viability, proliferation assessment, and migration

To evaluate the biocompatibility and proliferative effects of composite scaffolds, Live/Dead staining and CCK-8 assays were performed using material extracts from five experimental groups: Control, CSK@P100, CSK@P200, CSK@P300, and CSK@P/H100 (n = 3 per group). rBMSCs (2 × 10^4^ cells) were seeded in 1 mL of scaffold extract medium in 24-well plates and incubated for 24 h. For Live/Dead staining using Calcein AM/PI Double Staining Kit (1:100, E-CK-A354, Elabscience), cells were incubated in staining solution under dark conditions, washed three times with PBS, and visualized under fluorescence microscopy to assess cell viability in response to material extracts. For proliferation assessment, CCK-8 reagent (10 μL) was added to each well followed by 2-h incubation at 37 °C, and supernatants were transferred to 96-well plates for absorbance measurement at 450 nm using a microplate reader (BioTek Instruments, USA). Long-term proliferation analysis was conducted with the same extract-based protocol at days 1, 4, and 7 to evaluate sustained cellular growth in the presence of scaffold-derived compounds.

#### 2.6.3 Cell adhesion, migration, and recruitment assessment

To evaluate cell adhesion and morphology on scaffolds, rBMSCs were seeded onto scaffolds at a density of 2 × 10^4^ cells per well and cultured in 24-well plates with 1 mL scaffold extract medium for 24 h. Control group was cultured in regular culture medium without scaffold extracts. Cells were fixed with 4 % paraformaldehyde solution for 20 min at 4 °C. Cell permeabilization was performed using 0.2 % Triton X-100 solution for 4 min. Subsequently, rBMSCs were stained with FITC-labeled phalloidin solution (1:100, RM02836, ABclonal) to visualize F-actin cytoskeleton and incubated at room temperature for 20 min. Before each step, the previous working solution was removed and cells were gently washed with PBS. Finally, cell nuclei were stained with 4′,6-diamidino-2-phenylindole (DAPI, RM02978, ABclonal) for 5 min at room temperature. The stained cells were observed under a fluorescence microscope to assess cell adhesion, spreading, and cytoskeletal organization.

For the scratch wound healing assay, cells (2 × 10^5^ cells per well) were seeded in 6-well plates and cultured in material extract medium until reaching confluence. A uniform scratch was created using a 1 ml pipette tip, and wound closure was monitored at 0, 12, and 24 h time points to assess healing progression and quantified using ImageJ software.

For the FITC-based cell exclusion zone migration assay, a sterilized PDMS strip (0.5 cm wide) was placed centrally on the culture plate. Cells (≈1 × 10^5^ cells cm^−2^) were seeded on one side and cultured in material extract medium for 12 h. After adhesion, the PDMS strip was removed to create a cell-free migration zone. Cells were cultured in serum-free material extract medium for 48 h, then stained with FITC-labeled phalloidin solution for F-actin visualization. Migration was observed by CLSM (TCS SP8, Leica) and quantified using ImageJ software.

Cell recruitment performance was evaluated using Transwell migration assays (3422, Costar). Materials were placed in the lower chamber, and 3 × 10^4^ rBMSCs were seeded in the upper chamber. After 24 h of culture in α-MEM medium, the upper chamber was fixed with 4 % paraformaldehyde and stained with crystal violet (Beyotime, Shanghai, China). Non-penetrated cells were removed with cotton swabs, and migrated cells on the lower surface were observed under optical microscopy (Olympus) and quantified using ImageJ software.

#### 2.6.4 Alkaline phosphatase quantitative analysis (ALP) and alizarin red staining

The three types of scaffolds (Control, PCL, and PCL/HA) were placed in 24-well plates, and rBMSCs (2 × 10^5^ cells/well) were seeded onto the scaffold surfaces (n = 3), ensuring complete immersion in rBMSC osteogenic differentiation medium (OriCell, Guangzhou, China). On days 7 and 14, scaffolds were removed from the wells, and the well bottoms were stained using an ALP staining kit (Beyotime, Shanghai, China) to evaluate ALP activity. On days 14 and 21, scaffolds were removed and the well bottoms were additionally stained using an osteoblast mineralization nodule staining kit (Alizarin Red S method) (Beyotime, Shanghai, China) to assess calcium salt deposition. The results were observed and documented using an optical microscope.

#### 2.6.5 Real-time quantitative PCR (RT-qPCR) analysis of osteogenic and chondrogenic genes

*In vitro* studies, rBMSCs were seeded onto scaffolds and cultured in osteogenic differentiation medium for osteogenic analysis, with Control group and PCL and PCL/HA scaffold groups (n = 3 each). For chondrogenic analysis, rBMSCs were seeded onto scaffolds (CSK@P100, CSK@P200, CSK@P300, and CSK@P/H100) and cultured in chondrogenic differentiation medium (n = 3). We analyzed osteogenesis-related genes (*Alp*, bone morphogenetic protein 2 (*Bmp-2*), and osteocalcin (*Ocn*)) and chondrogenesis-related genes (proteoglycan 4 (*Prg4*), collagen type II alpha 1 chain (*Col2a1*), and collagen type X alpha 1 chain (*Col10a1*)).

On days 7 and 14, total RNA was extracted for gene expression analysis. For both osteogenic and chondrogenic groups, RNA was extracted from cells both on scaffolds and on culture plate surfaces using Trizol reagent (TaKaRa, Japan). RNA was reverse-transcribed using PrimeScript™ RT kit (TaKaRa, Japan) and analyzed by RT-qPCR using QSYBR Green Supermix on QuantStudioTM 7 Flex system. *Gapdh* served as housekeeping gene, with relative expression calculated using 2^-^ΔΔ^Ct method. *In vivo* studies, newly formed cartilage tissue was analyzed for *Prg4* and *Col10a1* expression using identical methodology. Primer sequences are listed in Table S1.

#### 2.6.6 Safranin O and alcian blue staining

The four types of cartilage layer scaffolds (CSK@P100, CSK@P200, CSK@P300, and CSK@P/H100) were placed in 24-well plates, and rBMSCs (1 × 10^5^ cells/well) were seeded onto the scaffold surfaces, ensuring complete immersion in culture medium. The cell-scaffold constructs were cultured in rBMSC chondrogenic differentiation medium (Solarbio, Beijing, China) for 24 days. To comprehensively evaluate extracellular matrix (ECM) production during chondrogenic differentiation, two complementary staining approaches were employed. First, scaffolds were stained with Safranin O solution (Beyotime, Shanghai, China) to visualize glycosaminoglycans within the scaffold matrix. Additionally, after removing the culture medium, Alcian Blue staining solution (Beyotime, Shanghai, China) was applied to the bottom of the well plates to assess ECM components that were secreted by the cells but not retained within the scaffolds, thus depositing onto the well surface. This dual staining strategy allowed for a more comprehensive evaluation of both scaffold-retained and secreted ECM production during the chondrogenic differentiation process. All staining results were observed and documented using a stereomicroscope.

### 2.7 Ectopic animal experiments (subcutaneous implantation)

#### 2.7.1 Chondrogenic differentiation of scaffold layers

The use of New Zealand white rabbits in this study was approved by the Animal Care and Use Ethics Committee of Jilin Province and conducted in accordance with the Guide for the Care and Use of Laboratory Animals (NIH Publication No. 8023, revised 1978). Additionally, the *in vivo* experiments were approved by the Laboratory Animal Ethics Committee of the School of Basic Medical Sciences, Jilin University (2024612). Three three-month-old New Zealand white rabbits (weighing 3 ± 0.5 kg) were randomly divided into four groups: CSK@P100, CSK@P200, CSK@P300, and CSK@P/H100 (n = 3 per group). The detailed grouping design for subcutaneous implantation is presented in Table 2. After anesthesia, different monolayer composite scaffolds loaded with rBMSCs (1 × 10^6^ cells) were implanted subcutaneously into the rabbits. To minimize individual differences, each rabbit was implanted with four different types of scaffolds. During the experimental period, penicillin (2 mg/kg) was injected daily for the following three days. At 8 weeks post-surgery, the rabbits were euthanized by air injection through the ear vein, and tissue samples were collected for immunofluorescence (IF) staining of Proteoglycan 4 (PRG4), Collagen II, and Collagen X. The fluorescence intensity was presented as topographic maps and semi-quantitatively analyzed.

#### 2.7.2 IF staining and semi-quantitative analysis of fluorescence intensity

IF staining was performed to evaluate cartilage-related protein expression in both monolayer scaffolds and newly formed cartilage tissue from animal studies. Samples were fixed in 4 % paraformaldehyde for 15 min at room temperature, permeabilized with 0.5 % Triton X-100 in PBS for 10 min, and blocked with 5 % bovine serum albumin (BSA) in PBS for 1 h at room temperature. Primary antibodies were diluted to their optimized concentrations: PRG4 (bs-11175R, Bioss, China), Collagen II (bs-10589R, Bioss, China), and Collagen X (bs-42277R, Bioss, China), and incubated overnight at 4 °C. After three washes with PBS, samples were incubated with Cy3-conjugated goat anti-rabbit IgG secondary antibody (1:200, Servicebio, China) for 1 h at room temperature in the dark. Cytoskeleton and nuclei were counterstained with Rhodamine-phalloidin (ab176757, Abcam, UK) for 30 min and DAPI (RM02978, ABclonal, China) for 5 min, respectively. Following final washes, samples were mounted with anti-fade mounting medium and examined under fluorescence microscopy using appropriate filter sets.

### 2.8 In situ animal experiments (articular cartilage defect model)

The use of New Zealand white rabbits in this study was approved by the Animal Care and Use Ethics Committee of Jilin Province and conducted in accordance with the Guide for the Care and Use of Laboratory Animals (NIH Publication No. 8023, revised 1978). Additionally, the *in vivo* experiments were approved by the Laboratory Animal Ethics Committee of the School of Basic Medical Sciences, Jilin University (2024612). Three-month-old New Zealand white rabbits (weighing 3 ± 0.5 kg) were randomly divided into four groups (n = 10 per group): S group (uniform pore-size scaffold), G group (gradient pore-size scaffold), CSK@S group, and CSK@G group. The detailed grouping design is presented in Table 3. As previously described, CSK was infiltrated into the cartilage regions of scaffold S (CSK@S) and scaffold G (CSK@G), establishing a comprehensive experimental matrix for comparative analysis. The rabbits were anesthetized with 3 % pentobarbital sodium (60 mg/kg). Cylindrical osteochondral defects (5 mm in radius and 5 mm in depth) were created in the central trochlear groove of the femur using an electric drill. The corresponding scaffolds were then implanted into the defects, and the rabbits received intramuscular injections of penicillin (2 mg/kg) once daily for three consecutive days. At weeks 6 and 12, the rabbits were euthanized by air embolism through the ear vein, and the distal femur samples were collected and preserved in 4 % paraformaldehyde.

#### 2.8.1 Micro-computed tomography (Micro-CT)

Subchondral bone regeneration in all experimental groups was evaluated using Micro-CT. Samples were scanned using a MicroCT42 system (SCANCO Medical, Switzerland) at 48 kV voltage, 200 μA current, and 18 μm pixel size, followed by three-dimensional image reconstruction. The images of regions of interest and osteochondral defect areas were analyzed using CTAn software. Multiple parameters were measured from the reconstructed images, including bone mineral density (BMD), bone volume fraction (BV/TV), and trabecular thickness (Tb.Th).

#### 2.8.2 Histological evaluation and safety assessment

Tissue samples were fixed in 4 % paraformaldehyde solution for 2 weeks, followed by decalcification in ethylenediaminetetraacetic acid (EDTA) solution at pH 7.4 for 12 weeks (EDTA supplied by Servicebio, China). The samples were then dehydrated and embedded in paraffin, and sectioned at 4 μm thickness. Hematoxylin and eosin (H&E) staining was performed to observe tissue morphology of the osteochondral repair sites. Additionally, at the 12-week time point, blood samples were collected via venipuncture for comprehensive hematological analysis, including complete blood count (CBC) and key biochemical indicators of hepatic and renal function. Subsequently, major organs including heart, liver, lung, kidney, and spleen were harvested and processed for H&E staining to evaluate potential systemic effects and biocompatibility of the implanted materials. Safranin O/Fast Green staining was used to visualize glycosaminoglycan (GAG) distribution and deposition in cartilage, as well as collagen content in subchondral bone. Furthermore, immunohistochemical staining was conducted using Collagen type I antibody (Collagen I, bs-10423R, Bioss, China) and Collagen II antibody (bsm-33409M, Bioss, China) to evaluate bone and cartilage-specific matrix formation, respectively. Heat-mediated antigen retrieval was performed using Tris/EDTA buffer (pH 9.0) prior to immunohistochemical staining. All sections were photographed using a bright-field microscope and analyzed using Case Viewer software (3DHISTECH, Budapest, Hungary) for comprehensive assessment of osteochondral tissue repair and systemic biocompatibility.

### 2.9 Immunohistochemical quantitative analysis

Immunohistochemical staining images were captured using a light microscope (Axio Scope.A1, Carl Zeiss, Oberkochen, Germany). The mean optical density (MOD) of Collagen II and Collagen I positive staining was quantified using ImageJ software (Version 1.53, National Institutes of Health, USA). For each sample, three random fields of view were selected from the cartilage repair region under the same magnification. The MOD values were calculated as integrated optical density (IOD) divided by the total area of the selected region.

### 2.10 Western blot (WB)

At 12 weeks postoperatively, newly formed cartilage tissue samples were collected for protein extraction using RIPA lysis buffer (P0013B, Beyotime, China) and quantified with a BCA protein assay kit (P0012, Beyotime, China). Proteins were separated by SDS-PAGE using precast gels (PG113, YSbio, China) and transferred onto PVDF membranes (88518, ThermoFisher, USA).

After blocking with rapid blocking buffer (PS108P, YSbio, China), membranes were incubated overnight at 4 °C with primary antibodies, followed by 1.5-h incubation with secondary antibodies at room temperature. Protein bands were visualized using ECL chemiluminescence detection kit (SQ202, YSbio, China) and captured with a ChemiDoc imaging system (Bio-Rad, USA).

Primary antibodies included β-actin (66009-1-Ig, Proteintech, USA), PRG4 (25732-1-AP, Proteintech, USA), BMP2 (abs147824, Absin, UK), DLX3 (13261-3-AP, Proteintech, USA), Collagen X (26984-1-AP, Proteintech, USA), and OSX (28694-1-AP, Proteintech, USA).

### 2.11 Statistical analysis

All experimental data were derived from at least three independent experiments and expressed as mean ± standard deviation (SD). Image analysis was performed using ImageJ software (National Institutes of Health, Bethesda, MD, USA). Statistical analysis was performed using one-way analysis of variance (ANOVA) followed by Tukey's multiple comparison test using GraphPad Prism 9 and Origin 2024 software. Differences were considered statistically significant when ∗*p* < 0.05, ∗∗*p* < 0.01, ∗∗∗*p* < 0.001, ∗∗∗∗*p* < 0.0001; and not significant (ns) when *p* > 0.05.

## 3 Results and discussion

### 3.1 Design and characterization of gradient osteochondral scaffolds

**Fig. 1:**
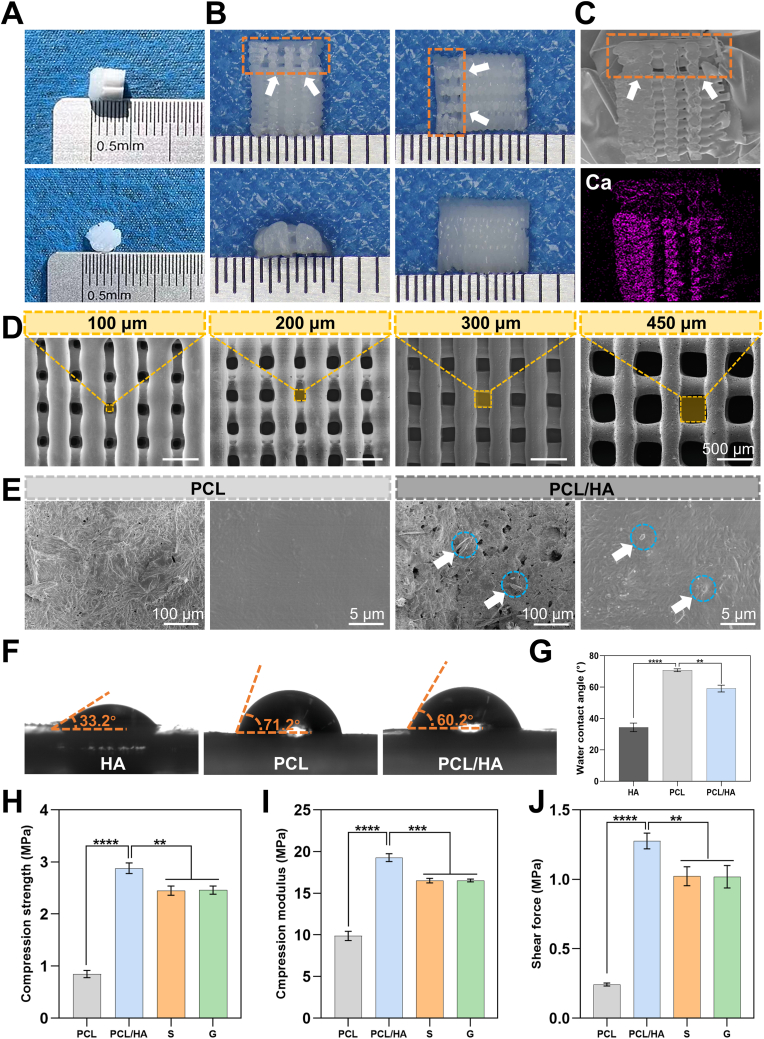
Comprehensive characterization of gradient composite osteochondral scaffolds. (A) Macroscopic view of complete scaffold structure. (B) Longitudinal cross-sections of gradient scaffolds under stereomicroscope showing gradient structure characteristics; cartilage regions highlighted (orange boxes). (C) Cross-sectional SEM image and Ca element mapping. (D) SEM images showing PCL scaffolds with varying pore sizes and PCL/HA scaffold; pores marked by yellow boxes. (E) High-magnification SEM images of PCL and PCL/HA scaffolds; HA particles indicated by blue circles. (F) Water contact angle measurements for HA, PCL, and PCL/HA. (G) Quantitative water contact angle analysis. (H–J) Mechanical properties: compression strength, compression modulus, and shear force of different scaffold compositions. (n = 3, ∗∗*p* < 0.01, ∗∗∗*p* < 0.001, ∗∗∗∗*p* < 0.0001).

As shown in Fig. 1A, the fabricated gradient scaffold was cylindrical with both diameter and height of 5 mm, featuring a translucent PCL portion at the top and a white PCL/HA portion at the bottom. As shown in Fig. 1B, we performed longitudinal sectioning of the gradient scaffold and observed the cross-sections from four aspects under stereomicroscope, clearly demonstrating the gradient characteristics of the scaffold, The orange rectangular box highlights the cartilage region of the scaffold. Additionally, we observed the longitudinal cross-sectional images using scanning electron microscopy (SEM) and conducted semi-quantitative analysis of calcium (Ca) elements through element mapping. The yellow rectangular box highlights the cartilage region of the scaffold (Fig. 1C). Energy dispersive X-ray spectroscopy (EDS) analysis revealed significantly increased Ca and phosphorus (P) content in the subchondral bone layer of the scaffold, creating a favorable mineralized environment for bone repair. Elemental mapping demonstrated uniform Ca distribution within this layer, confirming homogeneous HA incorporation into the PCL matrix and validating the scaffold's compositional gradient that mimics natural osteochondral tissue (Fig. S1) [25].

The gradient structure designed in this study draws inspiration from the natural osteochondral interface, particularly its progressive transition from cartilage to subchondral bone, and this bio-customized design is crucial for maintaining tissue function [26]. Unlike traditional homogeneous scaffolds [27,28], as shown in Fig. 1D, our five-layer gradient scaffold system featured: (1) superficial cartilage layer with 100 μm PCL pores facilitating chondrocyte adhesion, (2) middle cartilage layer with 200 μm PCL pores, (3) deep cartilage layer with 300 μm PCL pores promoting ECM secretion, (4) calcified cartilage transition layer with 100 μm PCL/HA pores providing biomineralization environment (Fig. S2), and (5) subchondral bone layer with 450 μm PCL/HA pores supporting bone formation. Top view and 3D perspective of different pore sizes are shown in Fig. S3. SEM analysis revealed distinct morphological characteristics between PCL and PCL/HA composites (Fig. 1e). The 100 μm PCL/HA calcified cartilage layer exhibited enhanced surface roughness with characteristic HA whisker structures and heterogeneous pore distribution, creating optimal microtopography for cartilage-bone interface formation. Blue circles highlight uniformly dispersed HA particles within the PCL matrix, confirming successful composite integration for the biomineralization process [29].

Comprehensive characterization was performed to validate successful PCL modification with HA, including FTIR spectroscopy (Fig. S4), hydrophilicity testing (Fig. 1F and G), and mechanical analysis (Fig. 1H–J). FTIR analysis revealed distinct chemical signatures among pure PCL, HA, and PCL/HA composites. The PCL/HA spectrum retained characteristic PCL peaks at 1724 cm^−1^ (C=O ester) and 2923 cm^−1^ (CH stretching), while exhibiting new HA-derived peaks at 1150 cm^−1^ and 960 cm^−1^ (PO_4_^3−^ symmetric and asymmetric stretching), confirming successful composite formation [30].

To evaluate the changes in surface hydrophilicity of the materials, water contact angle measurements were conducted on HA, PCL, and PCL/HA specimens (Fig. 1F and G). The results revealed that pure PCL exhibited a contact angle of 70.73 ± 0.9°, which significantly decreased to 59.07 ± 2.14° following the incorporation of 20 % HA into PCL, indicating enhanced hydrophilicity. In comparison, pure HA demonstrated a notably lower contact angle of 34.3 ± 2.72°, suggesting superior hydrophilic properties. These findings indicate that the introduction of HA not only improved the hydrophilicity of PCL but may also have enhanced material performance through increased surface roughness. The observed changes in contact angles were statistically significant (∗∗*p* < 0.01).

Mechanical analysis showed that HA incorporation significantly enhanced scaffold properties (Fig. 1H). PCL/HA scaffolds (2.88 ± 0.1 MPa) demonstrated superior compressive strength compared to pure PCL (0.85 ± 0.07 MPa, ∗∗∗∗*p* < 0.0001). Groups S and G showed intermediate values (∼2.45 MPa, *p* < 0.01 vs PCL), indicating that HA reinforcement effectively improved mechanical performance through stress distribution and polymer chain stabilization [31]. Consistent improvements were observed across all mechanical parameters (Fig. 1I and J).

In addition, comprehensive *in vitro* characterization of the CS-KGN system injected into the cartilage layers was performed to evaluate their degradation behavior and KGN release kinetics. CS hydrogels were immersed in PBS containing lysozyme (10 mg/mL) and incubated at 37 °C to simulate physiological conditions. The gross morphology of KGN-loaded CS hydrogels before and after gelation is presented in Fig. S5, while SEM analysis revealed the porous microstructure of the hydrogel network (Fig. S6). After 14 days, CS hydrogels retained 65.67 ± 1.16 % of their initial mass, demonstrating controlled degradation (Fig. S6A). KGN release exhibited a characteristic biphasic pattern with an initial burst release of 15.33 ± 1.53 % within 24 h, followed by sustained release reaching 66.33 ± 3.22 % total cumulative release over 14 days (Fig. S7B). This controlled release profile provides optimal drug delivery kinetics for sustained chondrogenic stimulation. Collectively, these results demonstrate the successful construction of a bio-customized gradient scaffold system CSK@G that recapitulates the hierarchical structure of the natural osteochondral interface.

### 3.2 In vitro cell viability, proliferation, stem cell migration and recruitment assessment

**Fig. 2:**
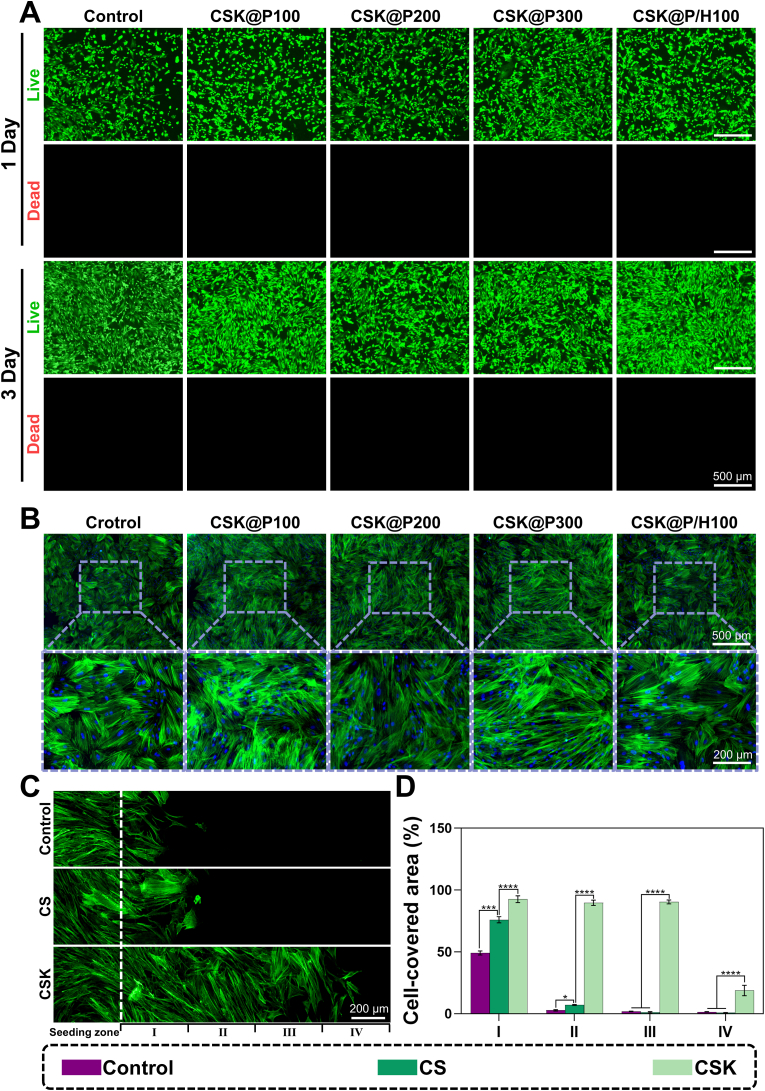
*In vitro* biocompatibility and stem cell migration capacity evaluation of composite scaffolds. (A) Live/dead staining of rBMSCs at 1 and 3 days (green: viable cells, red: dead cells). (B) Cytoskeletal organization of rBMSCs on scaffolds at day 3, stained with FITC-phalloidin (green) and DAPI (blue). (C) Stem cell migration showing cellular mobilization capacity. (D) Quantitative analysis of cell coverage across four migration zones (I-IV) showing percentage of cell-covered area for each treatment group. (n = 3, ∗*p* < 0.05, ∗∗∗*p* < 0.001, ∗∗∗∗*p* < 0.0001).

We conducted live/dead staining experiments to evaluate material cytotoxicity and biocompatibility. As shown in Fig. 2A, rBMSCs co-cultured with different groups for 1 and 3 days showed ideal cell survival in all groups, indicating good biocompatibility without cytotoxicity. Statistical analysis revealed no significant differences in cell survival rates between groups (*p* > 0.05) (Fig. S8A). Subsequently, FITC-phalloidin staining of rBMSCs after 3 days co-culture showed good cell adhesion and spreading in all groups under fluorescence microscopy, with no notable differences observed between groups (Fig. 2B).

We then used the CCK-8 assay to quantitatively evaluate the materials' effects on cell proliferation, observing cell growth after 1, 3, and 7 days of co-culture. As shown in Fig. S8B, rBMSCs demonstrated a gradual increase in proliferation over time. By day 7, compared to the Control group, the experimental groups showed significantly enhanced cell proliferation (∗∗*p* < 0.01). This enhancement can be attributed to the combined effects of CS and KGN: the CS hydrogel provides a favorable microenvironment for rBMSC adhesion and proliferation, while KGN promotes cell proliferation through its bioactive properties, together creating an optimal condition for cell growth. These results are consistent with previously reported biocompatibility characteristics of CS hydrogels [32].

Notably, our experimental results confirmed the exceptional biocompatibility of the CS-KGN hydrogel system and revealed its synergistic promotional effects on cellular behavior. The enhanced cell proliferation can be attributed to CS's molecular architecture, where amino and hydroxyl groups create an ECM-mimetic microenvironment facilitating cell adhesion and proliferation, while the porous network provides adequate space and nutrient diffusion pathways for cellular growth.

In the early stages of osteochondral healing, the homing, adhesion, and migration of rBMSCs constitute indispensable components of effective tissue regeneration, orchestrating the entire repair process from initial injury response to ultimate tissue restoration. Our experimental results demonstrate the critical role of material composition in facilitating these cellular processes.

To comprehensively evaluate cellular migration capacity, we employed both traditional scratch wound healing assays and a modified cell exclusion zone assay. Traditional scratch assays (Fig. S9A) with quantitative analysis (Fig. S9B) demonstrated that KGN exhibits significant stem cell migration-promoting capabilities, while CS also enhances migration to a certain extent, likely attributed to its favorable cell adhesion properties and biocompatibility.To avoid potential edge damage effects inherent in conventional scratch methods, we further employed a FITC-based cell exclusion zone migration assay (Fig. 2C and D). This assay reveals that KGN incorporation dramatically enhances stem cell migration, demonstrating its dual functionality in cellular mobilization and proliferation enhancement. This migration enhancement is dramatically amplified in KGN-loaded scaffolds, indicating significantly improved cellular mobilization properties. These two distinct migration assays collectively provide robust validation of the superior cellular motility-promoting effects of the KGN-CS system.Additionally, our Transwell recruitment assay (Fig. S10A) reveals that while CS does not exhibit significant stem cell recruitment capacity, KGN-containing groups demonstrate significantly enhanced stem cell recruitment capacity, with quantitative analysis (Fig. S10B) providing statistical confirmation of these recruitment findings.

Moreover, KGN demonstrates an excellent biosafety profile that establishes a crucial foundation for clinical translation. Previous studies have confirmed that KGN exhibits dose-dependent biological effects [33], and our systematic optimization research [24] has identified the optimal concentration that achieves maximum biological activity while maintaining cellular viability. This careful balance represents a critical consideration for developing clinically viable tissue engineering constructs.

Collectively, these findings highlight the pivotal role of CS-KGN hydrogel systems in creating an optimal microenvironment that facilitates cellular recruitment, adhesion, and migration. The combined effects observed between the CS and KGN incorporation provide compelling evidence supporting the therapeutic potential of this biomaterial system in cartilage tissue engineering applications.

### 3.3 In vitro evaluation of osteogenic differentiation of rBMSCs on subchondral scaffolds

We conducted a comprehensive evaluation of the osteogenic potential of the subchondral bone layer scaffolds through ALP staining, ARS staining, and expression analysis of osteogenesis-related genes (*Alp*, *Bmp-2*, and *Ocn*) to investigate the effects of these scaffolds on rBMSCs osteogenic differentiation. The experimental design consisted of three groups: Control group (rBMSCs cultured in osteogenic differentiation medium only), PCL group (rBMSCs seeded onto PCL scaffolds), and PCL/HA group (rBMSCs seeded onto PCL/HA composite scaffolds). For ALP and ARS staining analysis, scaffolds were removed and staining was performed on cells adhered to the culture plate bottom.

**Fig. 3:**
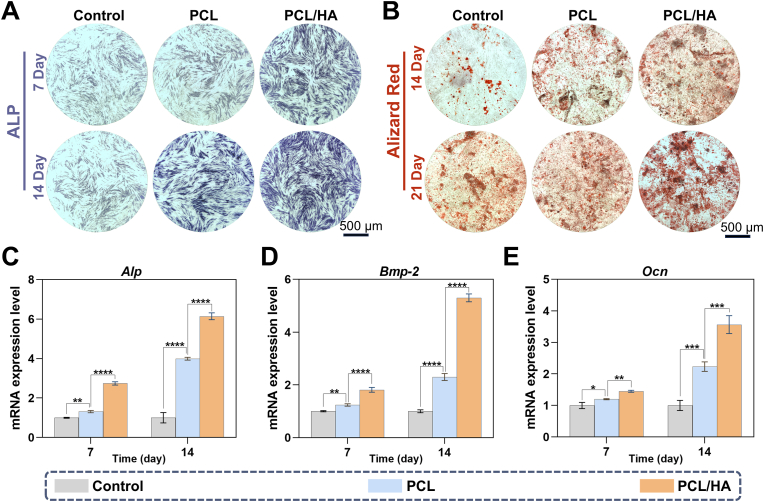
*In vitro* osteogenic activity in Control, PCL, and PCL/HA scaffold groups. (A) ALP staining after 7 and 14 days of osteogenic induction. (B) ARS staining after 14 and 21 days of osteogenic induction. (C–E) mRNA expression levels of *Alp*, *Bmp-2*, and *Ocn* after 7 and 14 days of osteogenic induction. (n = 3, ∗*p* < 0.05, ∗∗*p* < 0.01, ∗∗∗*p* < 0.001, ∗∗∗∗*p* < 0.0001).

After 7 and 14 days of co-culture, the PCL/HA group exhibited more intense ALP staining (Fig. 3A), indicating enhanced early osteogenic activity. Furthermore, after 14 and 21 days of culture, ARS staining revealed that the PCL/HA group showed the highest level of mineralized matrix deposition, as evidenced by the most intense staining (Fig. 3B). Notably, even in the absence of the osteoinductive component HA, the PCL group demonstrated improved osteogenic differentiation compared to the control group, likely due to the intrinsic porous structure of PCL, which facilitates the osteogenic differentiation of rBMSCs. This observation was further corroborated by RT-qPCR analysis. RT-qPCR analysis of osteogenic gene expression, including *Alp*, *Bmp-2*, and *Ocn* (Fig. 3C–E), revealed that the PCL/HA group exhibited significantly upregulated expression of these genes on day 7. By day 14, the PCL/HA group maintained significantly higher levels of osteogenic gene expression compared to the other groups. These results collectively demonstrate the excellent osteoinductive properties of the PCL/HA composite scaffold, which may be attributed to several factors: 1) HA, as a primary component of natural bone mineral, mimics the inorganic environment of bone tissue; 2) The presence of HA enhances scaffold surface bioactivity, providing additional cell attachment sites; 3) HA may activate osteogenic pathways through the release of calcium and phosphate ions.

These results demonstrate a synergistic effect: the porous PCL structure provides a conducive environment for osteogenic differentiation, while the incorporation of HA further enhances this osteogenic potential through bioactive mineral components [34,35].

### 3.4 In vitro evaluation of chondrogenic differentiation of rBMSCs on cartilage layer scaffolds

**Fig. 4:**
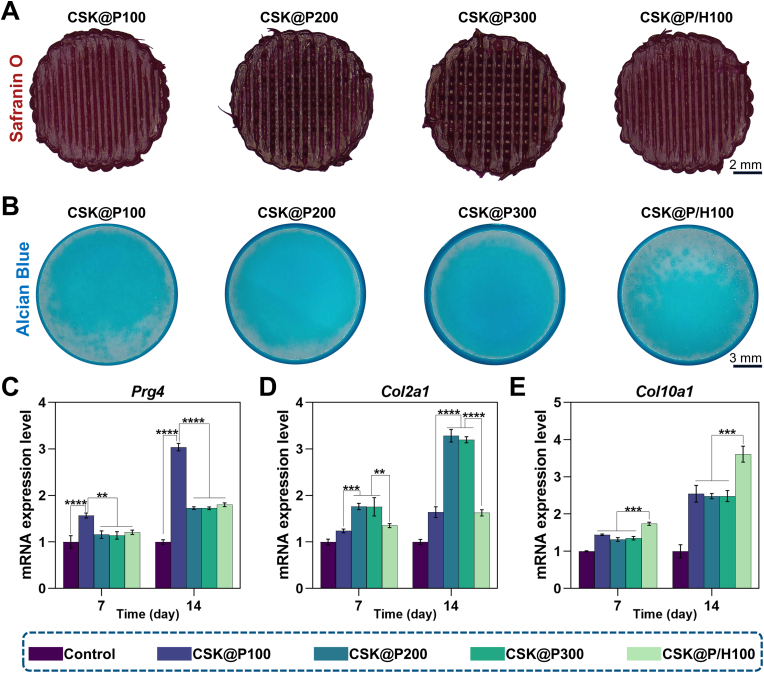
*In vitro* chondrogenic activity and cartilage matrix formation in CSK@P100, CSK@P200, CSK@P300, and CSK@P/H100 groups. (A) Safranin O staining after 24 days of culture on CSK@P100, CSK@P200, CSK@P300, and CSK@P/H100 surfaces. (B) Alcian Blue staining at the bottom of culture plates after 24 days of co-culture with CSK@P100, CSK@P200, CSK@P300, and CSK@P/H100. (C–E) mRNA expression levels of *Prg4*, *Col2a1*, and *Col10a1* after 7 and 14 days of chondrogenic induction. (n = 3, ∗∗∗*p* < 0.001, ∗∗∗∗*p* < 0.0001).

As previously described, the chondrogenic differentiation experiments were divided into four groups: CSK@P100, CSK@P200, CSK@P300, and CSK@P/H100. To assess the effects of scaffolds with different pore sizes on the chondrogenic differentiation of rBMSCs, Safranin O staining was performed after 24 days of co-culture. The results showed that the CSK@P200 and CSK@P300 groups exhibited the most intense staining (Fig. 4A). Consistently, Alcian blue staining of the bottom of the culture plates (Fig. 4B) was also most pronounced in the CSK@P200 and CSK@P300 groups. Based on the mechanisms of Safranin O and Alcian blue staining, these findings indicate that these two scaffolds markedly promoted the differentiation of rBMSCs into mid/deep zone chondrocytes and significantly enhanced the production and secretion of ECM. Notably, the intensified staining observed at the bottom of the plates suggests that the ECM secreted by cells on these scaffolds was highly abundant, with excess matrix not retained by the scaffolds depositing onto the plate surface. This further highlights the superior performance of these scaffolds in promoting chondrogenic differentiation and matrix production.

To evaluate the differential effects of scaffold pore sizes on rBMSC differentiation into stratified chondrocyte phenotypes, we performed quantitative PCR analysis of layer-specific marker genes—*Prg4* (superficial), *Col2a1* (middle/deep), and *Col10a1* (calcified zones)—following 7 and 14 days of co-culture (Fig. 4C–E). CSK@P100 scaffolds demonstrated significantly elevated *Prg4* expression compared to all other groups (∗∗∗*p* < 0.001). Both CSK@P200 and CSK@P300 groups exhibited markedly higher *Col2a1* expression than remaining groups, with no significant inter-group difference (*p* > 0.05). Notably, CSK@P/H100 scaffolds showed the highest *Col10a1* expression, significantly surpassing all other conditions (∗∗∗∗*p* < 0.0001), consistent with our hypothesis.

These results reveal the significant influence of scaffold pore size on rBMSCs chondrogenic differentiation. Smaller pores (100 μm) appeared to favor superficial zone cartilage formation, possibly due to their dense structure mimicking the characteristics of superficial zone cartilage. Medium-sized pores (200–300 μm) promoted middle/deep zone cartilage formation, likely because this pore range better facilitates cell migration and nutrient diffusion. The CSK@P/H100 scaffold with hydroxyapatite significantly enhanced *Col10a1* expression, indicating its role in promoting chondrocyte hypertrophy and calcification, which aligns with the biological properties of hydroxyapatite. These findings provide new insights for designing tissue-engineered cartilage with zone-specific properties. Of course, we must acknowledge that we did not directly observe the differentiation of rBMSCs into specific cartilage cell types, such as mid/deep zone chondrocytes, superficial zone chondrocytes, or calcified cartilage layer cells. This conclusion is based on a hypothesis derived from the expression patterns of genes highly expressed in the superficial zone, mid/deep zone, and calcified cartilage layer, as well as the secretion characteristics and staining results observed during the experiments.

### 3.5 In vivo ectopic evaluation (subcutaneous section)

#### 3.5.1 In vivo ectopic evaluation of cartilage layer expression promoted by scaffolds with different pore sizes

**Fig. 5:**
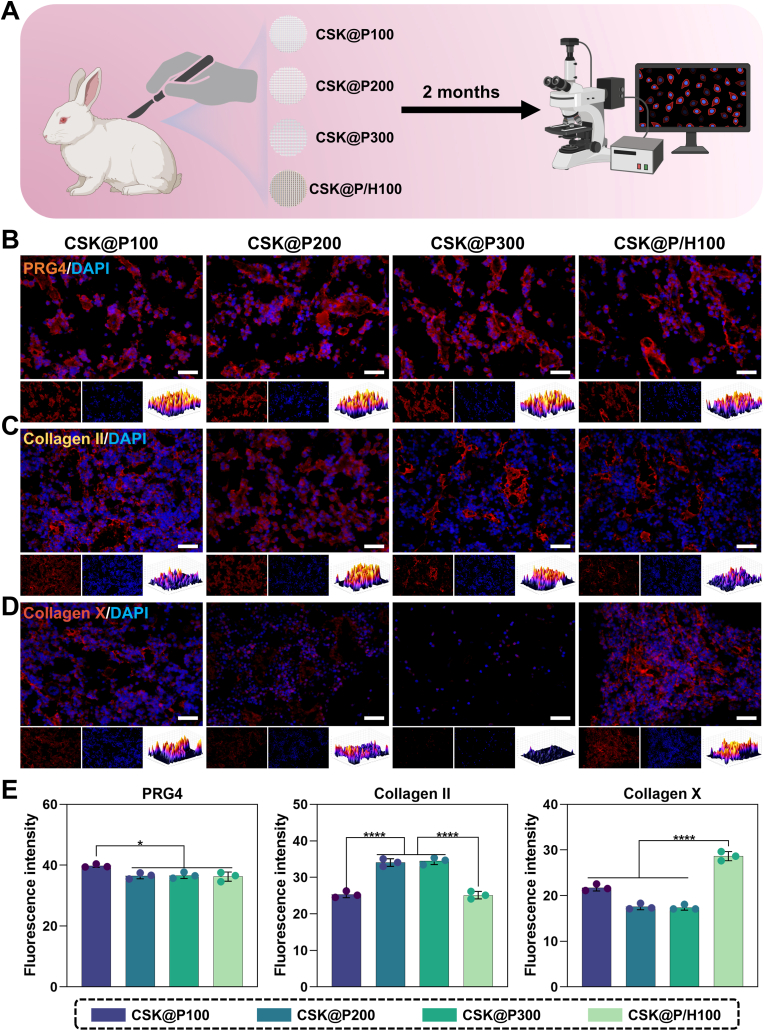
IF analysis of cartilage layer scaffolds (CSK@P100, CSK@P200, CSK@P300, and CSK@P/H100) retrieved 2 months after subcutaneous implantation. (A) Schematic illustration of subcutaneous implantation procedure. (B–D) Representative IF images of cartilage-related markers PRG4, Collagen II, and Collagen X (red), with cell nuclei shown in blue (DAPI), and topographical maps showing relative fluorescence intensity. (E) Quantitative statistical analysis of fluorescence intensity for PRG4, Collagen II, and Collagen X. (n = 3, ∗*p* < 0.05, ∗∗∗∗*p* < 0.0001, scale bars: 50 μm).

Prior to conducting *in vivo* orthotopic animal experiments (articular portion), we first performed an 2 months *in vivo* ectopic experiment (subcutaneous implantation) to validate the differential effects of four scaffolds with varying pore sizes on zonal cartilage differentiation, while minimizing unnecessary animal use and ensuring experimental rigor (Fig. 5A). Previous studies have demonstrated good consistency between *in vivo* ectopic and orthotopic experimental results [[36], [37], [38]]. The experimental groups remained consistent with the aforementioned setup.

By analyzing PRG4 IF images and visualizing fluorescence intensity as topographical maps, we found that the CSK@P100 group exhibited the highest fluorescence intensity (Fig. 5B). Further quantitative analysis of PRG4 fluorescence intensity revealed that the CSK@P100 group was significantly higher than the other three groups (∗*p* < 0.05) (Fig. 5E). Regarding Collagen II fluorescence intensity, CSK@P200 and CSK@P300 groups showed the strongest fluorescence (Fig. 5C), with statistical analysis indicating significantly higher intensity compared to the other two groups (∗∗∗∗*p* < 0.0001) (Fig. 5E). As the primary marker protein for calcified cartilage layer, Collagen X expression was strongest in the CSK@P/H100 group, showing significant statistical differences compared to the other three groups (∗∗∗∗*p* < 0.0001) (Fig. 5D and E).

These experimental results strongly support our initial design concept. The small pore structure (100 μm) of the CSK@P100 scaffold successfully mimicked the dense characteristics of the superficial layer of articular cartilage, providing an ideal microenvironment for high PRG4 expression. This structural feature effectively promoted the formation of superficial cartilage phenotype by influencing cell spatial arrangement and mechanical stimulation [[39], [40], [41]]. Meanwhile, the medium pore sizes (200–300 μm) of CSK@P200 and CSK@P300 scaffolds demonstrated excellent middle/deep layer cartilage induction capability. This pore size range not only facilitated cell migration and proliferation but also ensured better nutrient diffusion and metabolic waste removal, with structural features similar to the ECM conformation of natural cartilage tissue.

Notably, the addition of hydroxyapatite in the CSK@P/H100 scaffold significantly promoted Collagen X expression, indicating that hydroxyapatite successfully simulated the mineralized microenvironment of subchondral bone, providing necessary mineralization signals for the formation of calcified cartilage layer [42]. The *in vivo* subcutaneous experiments validated our hypothesis that the constructed cartilage layer scaffold could induce zonal cartilage regeneration, providing a reliable theoretical basis and experimental support for subsequent in situ *in vivo* experiments (articular experiments). These results not only confirmed the rationality of the scaffold design but also provided new insights and methods for constructing tissue-engineered cartilage with zonal structure. Although the above results effectively demonstrated that scaffolds with different pore sizes could guide stem cells to differentiate into chondrocytes with distinct zonal characteristics, we must acknowledge, consistent with the rationale applied in the *in vitro* chondrogenesis experiments, that we did not directly observe these specific differentiation events. This conclusion is inferred from the differential expression patterns of key proteins associated with distinct cartilage layers, as revealed by immunofluorescence analysis, which strongly supports the hypothesis of zonal cartilage regeneration.

### 3.6 In vivo orthotopic experiments (articular portion)

#### 3.6.1 Micro-CT evaluation of subchondral bone defect repair

**Fig. 6:**
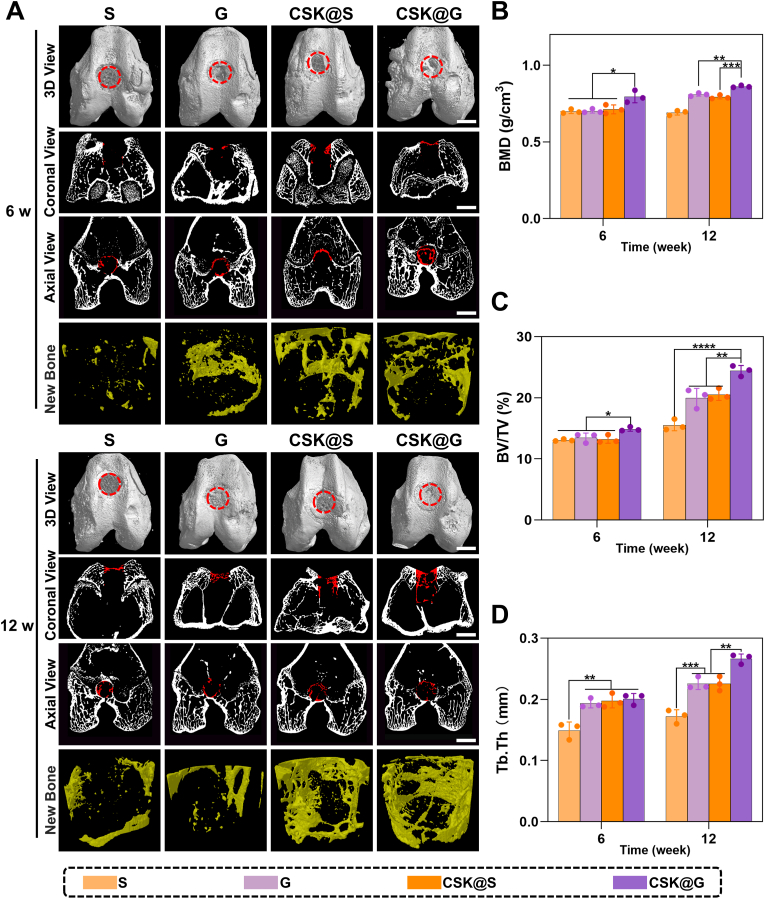
Micro-CT analysis of regenerated subchondral bone in defect sites of different groups at 6 and 12 weeks post-surgery. (A) 3D reconstruction images, 2D projection images in transverse view, longitudinal view, and 3D reconstruction images of regenerated bone from different groups. Red circles or rectangular regions indicate newly formed tissue. (B–D) Quantitative analysis of Micro-CT parameters (BMD, BV/TV, Tb.Th) in the defect area at 6 and 12 weeks post-surgery. (n = 3, ∗*p* < 0.05, ∗∗*p* < 0.01, ∗∗∗*p* < 0.001, ∗∗∗∗*p* < 0.0001, scale bars: 5 mm).

Fig. 6 demonstrates the repair of subchondral bone defects observed through Micro-CT at 6 and 12 weeks after *in vivo* orthotopic (articular) surgery. The intraoperative implantation procedure is shown in Fig. S11. In Fig. 6A, the 3D View shows the modeled defect site indicated by red circles; the red circular regions in the Coronal View and red rectangular regions in the Axial View indicate regenerated tissue, with white areas representing calcified tissue; New Bone shows the 3D reconstruction of newly formed bone tissue. Comprehensive analysis of key bone morphometric parameters including BMD, BV/TV, and Tb.Th revealed that the CSK@G group demonstrated significant advantages in promoting subchondral bone repair. As shown in Fig. 6B, the BMD values of the CSK@G group showed statistical differences compared to the other three groups at 6 weeks (*p* < 0.05), and by 12 weeks, the CSK@G group showed statistically significant differences compared to the CSK@S group (*p* < 0.001), fully demonstrating the excellent effectiveness of the gradient scaffold in subchondral bone repair. As illustrated in Fig. 6C–D, in the analysis of BV/TV and Tb.Th, whether during the short-term observation period at 6 weeks or the long-term evaluation at 12 weeks, the CSK@G group consistently exhibited optimal bone repair outcomes, with BV/TV and Tb.Th parameters significantly superior to other experimental groups, fully confirming the excellent performance and sustained stable therapeutic effects of this gradient composite material in subchondral bone defect repair.

Notably, in our study is that although KGN was used in the cartilage layer of the CSK@S group, some KGN might have been released into the subchondral bone layer, thereby promoting subchondral bone repair [43,44]. Nevertheless, the G group without KGN and the CSK@S group with KGN showed no significant statistical differences across multiple parameters. Previous studies have shown that trace amounts of KGN may have certain promoting effects on subchondral bone repair [45,46]. This finding further suggests that the multilayer porous scaffold constituting the cartilage layer, when combined with KGN, could achieve the same promoting effect on subchondral bone repair as KGN alone. Another surprising finding is that despite using consistent structures in the subchondral bone layer, Micro-CT scanning and statistical analysis results showed that the CSK@G group still achieved the best subchondral bone repair outcomes. Besides KGN release promoting subchondral bone repair, the scaffold structure in the CSK@G group might have played a more active role in the repair process [47]. This may also be attributed to the secretion of various factors during cartilage repair, such as Transforming Growth Factor-β (TGF-β) and Insulin-like Growth Factor (IGF), which not only promote cartilage growth and repair but may also penetrate into bone tissue, promoting subchondral bone formation [[48], [49], [50]]. Additionally, cartilage and bone are structurally closely connected, with a biomechanical coupling relationship between them. Better cartilage repair typically indicates better cartilage layer integrity, thus reducing the direct transmission of external impacts or loads to the subchondral bone layer. This shock-absorbing effect may, to some extent, protect the subchondral bone layer, indirectly promoting subchondral bone repair [51]. It can be understood that the cartilage repair outcome influences the subchondral bone repair to some extent [52,53]. This further validates the optimal performance of the CSK@G group in cartilage repair.

#### 3.6.2 Histological evaluation of osteochondral repair

**Fig. 7:**
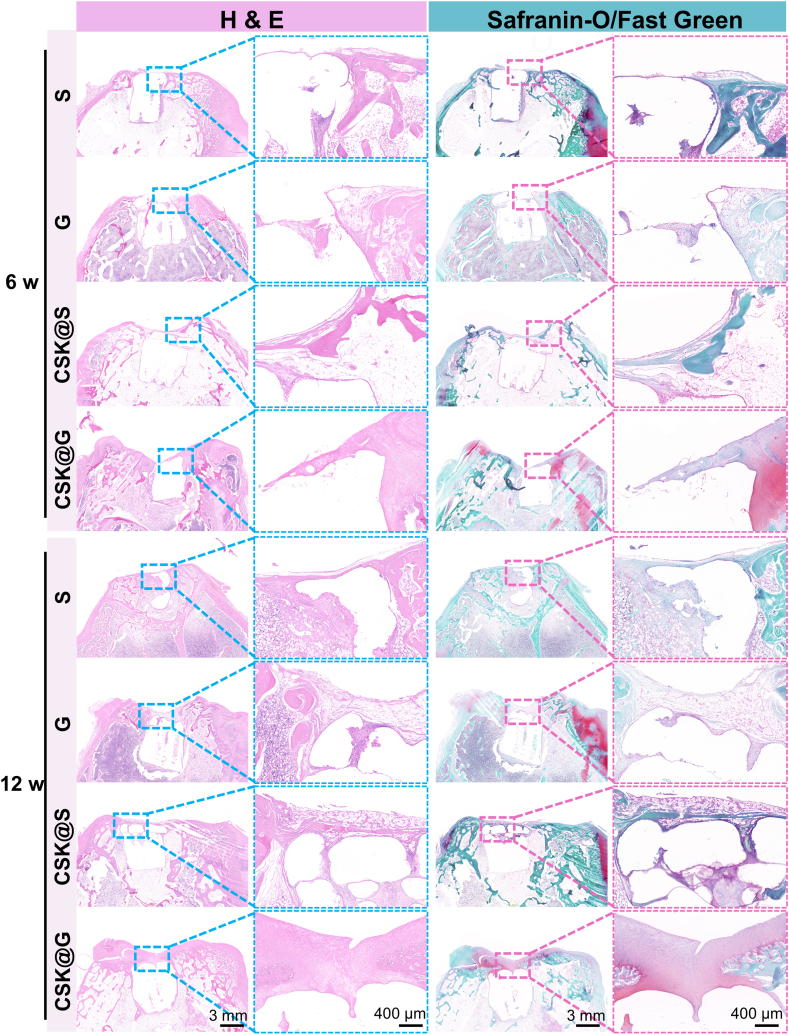
H&E and Safranin O-Fast Green staining of defect sites in different groups at 6 and 12 weeks post-surgery. (n = 3) (Boxes indicate newly formed tissue in the cartilage layer and boundaries).

**Fig. 8:**
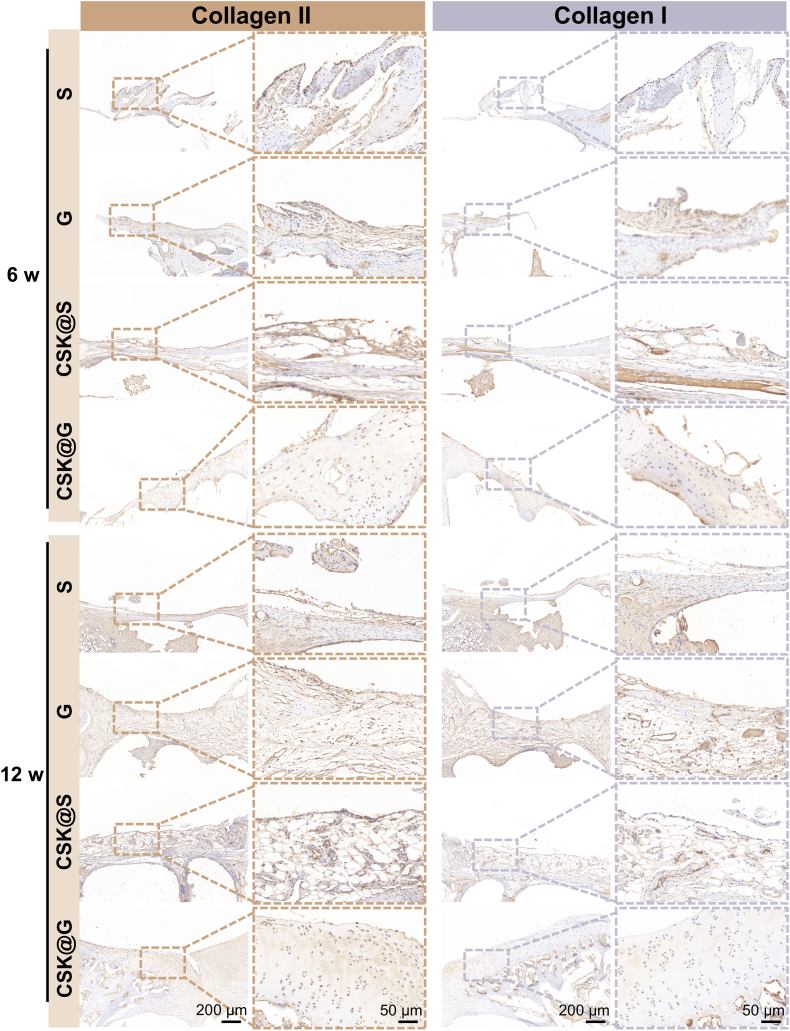
Immunohistochemical staining of Collagen II and Collagen I in defect sites of different groups at 6 and 12 weeks post-surgery. (n = 3) (Boxes indicate newly formed tissue in the cartilage layer and boundaries).

Histological staining analysis provided crucial evidence for in-depth evaluation of CSK@G scaffold's role in articular cartilage and subchondral bone repair. Through complementary staining techniques of H&E, Safranin O-Fast Green, and immunohistochemical staining for Collagen II and Collagen I, we systematically observed and analyzed the repair outcomes across different experimental groups at 6 and 12 weeks (Fig. 7, Fig. 8). H&E staining results comprehensively demonstrated the formation process and quality of newly formed tissue at the osteochondral defect site following scaffold implantation. Results indicated that the CSK@G group exhibited significantly superior repair in both cartilage and subchondral bone compared to other control groups (S, G, and CSK@G group). This finding highly correlated with previous Micro-CT scanning data, further validating the significant advantages of the CSK@G group in tissue structure reconstruction and bone density restoration. Remarkably, at 12 weeks post-implantation, both HE and Safranin O-Fast Green staining under high magnification revealed that the regenerated cartilage in the CSK@G group displayed distinct chondrocyte morphological features with well-defined lacunar structures, which represents a particularly encouraging outcome. The presence of these characteristic lacunae, housing individual chondrocytes, demonstrates successful recapitulation of native cartilage architecture and indicates mature cartilage tissue formation rather than merely fibrous tissue repair, with Safranin O-Fast Green staining further confirming abundant proteoglycan deposition (Fig. S12).

In the detailed analysis of cartilage repair, the CSK@G group demonstrated significantly superior tissue regeneration capability compared to other experimental groups. Specifically, the newly formed cartilage in this group exhibited not only high morphological regularity but also ideal microscopic density, collectively indicating excellent cartilage regeneration potential [54].

Through systematic observation using Safranin O-Fast Green specific staining technique and immunohistochemical analysis of Collagen II and Collagen I, we were able to reveal more detailed microscopic aspects of cartilage repair: firstly, at the interface between the repair region and surrounding native cartilage tissue, a naturally transitioning and structurally continuous integration zone was formed, which is crucial for ensuring long-term stability of the repaired tissue; secondly, within the cartilage layer, abundant newly formed cartilage tissue with intact morphology and ordered arrangement was observed, with strong Collagen II expression indicating successful chondrogenic differentiation, while controlled Collagen I expression suggested appropriate tissue maturation without excessive fibrosis. Quantitative analysis of MOD further confirmed these findings (Fig. S13): the CSK@G group exhibited significantly higher Collagen II expression and lower Collagen I expression compared to other groups, demonstrating successful scaffold-induced directional differentiation of chondrocytes and tissue reconstruction [55].

In comparative analysis across all experimental groups, the CSK@G group demonstrated several notable advantages in repair outcomes: the thickness of the repaired cartilage approached that of normal cartilage tissue, tissue continuity was well maintained, and near-perfect structural integration with adjacent native cartilage tissue was achieved [56]. This high-quality repair was evident not only in macroscopic morphology but, more importantly, also exhibited histological characteristics highly similar to natural cartilage at the microscopic structural level. In contrast, other experimental groups (S group, G group, and CSK@S group) showed significant limitations in their repair outcomes: firstly, the thickness of repaired cartilage was generally below normal levels, indicating insufficient cartilage tissue regeneration; secondly, tissue continuity showed obvious defects, manifesting as local disruption or loosening; lastly, obvious structural breaks and discontinuities were observed in the transition zone with normal cartilage, and such abrupt interface transitions might lead to decreased mechanical properties of the repaired tissue and increase the risk of subsequent degeneration [57].

These compelling observations not only substantiated the exceptional efficacy of the CSK@G scaffold in promoting cartilage regeneration from a histological standpoint but also unveiled critical mechanistic insights: the meticulously engineered gradient architecture likely orchestrates superior tissue regeneration by establishing a bio-customized microenvironment that optimally supports chondrocyte adhesion, proliferation, and phenotypic differentiation. Importantly, comprehensive systemic safety assessment via histopathological examination of vital organs (heart, liver, lung, spleen, and kidney) at 12 weeks post-implantation revealed no detectable pathological alterations, thereby establishing the exceptional biocompatibility and non-cytotoxic profile of our engineered scaffold system (Fig. S14). Furthermore, hematological and biochemical analyses confirmed systemic safety with red blood cells (RBC), white blood cells (WBC), and hepatorenal function indicators including aspartate aminotransferase (AST) and creatinine (Cr) remaining within normal physiological ranges, demonstrating excellent biocompatibility with negligible systemic toxicity throughout the healing period (Fig. S15). These multifaceted findings provide pivotal translational implications for the rational design optimization of next-generation scaffolds and the advancement of precision cartilage repair therapeutics [58,59].

#### 3.6.3 Evaluation of chondrogenic and ossification markers in the superficial layer of cartilage tissue by IF staining, RT-qPCR analysis, and WB

**Fig. 9:**
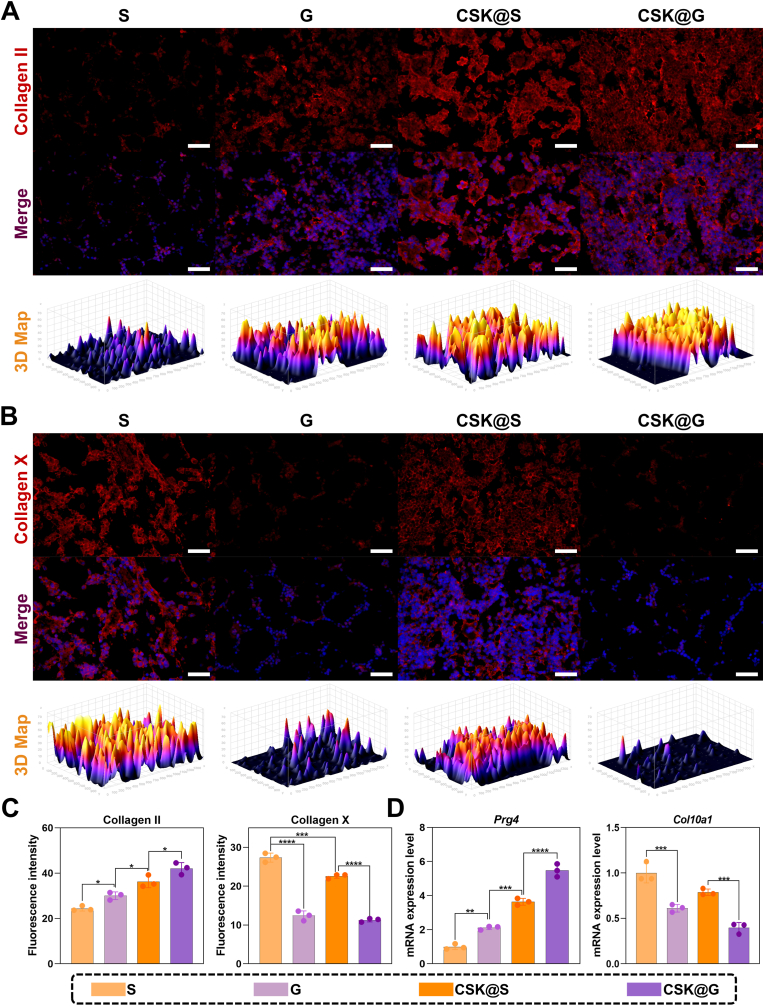
Evaluation of chondrogenic and ossification of the superficial layer by IF staining and RT-qPCR analysis of different groups after 12 weeks of surgery. (A) IF staining of Collagen II (red). (B) IF staining of Collagen X (red). (C) Semi-quantitative analysis of fluorescence intensity. (D) Quantitative RT-qPCR analysis of *Prg4* and *Col10a1* gene expression in newly formed cartilage layer. (n = 3, ∗*p* < 0.05, ∗∗*p* < 0.01, ∗∗∗*p* < 0.001, ∗∗∗∗*p* < 0.0001, scale bars: 50 μm).

Given that cartilage regeneration presents greater challenges than subchondral bone repair, and regenerated cartilage is particularly susceptible to ossification, evaluating the quality and phenotypic stability of regenerated cartilage tissue is crucial. Therefore, we comprehensively assessed the quality of newly formed cartilage and its ossification tendency at 12 weeks post-surgery through IF and RT-qPCR analyses (Fig. 9), as well as WB analysis (Fig. S16).

IF analysis revealed significantly enhanced Collagen II expression in CSK@S and CSK@G compared to S and G (Fig. 9A), indicating that CS-KGN incorporation markedly promoted cartilage matrix formation and glycosaminoglycan deposition. Three-dimensional fluorescence intensity mapping demonstrated more uniform and intense Collagen II distribution in CS-KGN-modified scaffold groups, confirming that the CS-KGN system facilitated superior cartilaginous differentiation and cartilage-specific matrix synthesis.

Conversely, Collagen X IF staining revealed distinct differences among groups. The S and CSK@S exhibited higher Collagen X expression in the superficial cartilage layer, suggesting undesirable calcification, which may be attributed to the uniform pore structure of conventional scaffolds promoting further ossification following initial cartilage formation. In contrast, the G and CSK@G showed significantly reduced Collagen X expression in the superficial cartilage layer, indicating that gradient scaffold design better preserved cartilage phenotype and effectively prevented newly formed cartilage calcification. Semi-quantitative fluorescence intensity analysis (Fig. 9C) confirmed significantly elevated Collagen II expression in the CSK@G (∗*p* < 0.05), while Collagen X levels in S and CSK@S were higher than those in G and CSK@G, preliminarily confirming the advantage of gradient scaffolds in preventing newly formed cartilage calcification.

Furthermore, scaffold groups without CSK hydrogel systems exhibited more pronounced ossification tendencies, potentially related to the absence of sustained biological and physical stimulation. These results demonstrate that the synergistic effect of gradient pore architecture and hydrogel delivery system creates an optimal microenvironment for cartilage regeneration, effectively maintaining cartilage homeostasis and preventing cartilage ossification.

Quantitative RT-qPCR analysis (Fig. 9D) further corroborated these IF findings, with the CSK@G exhibiting the highest *Prg4* and lowest *Col10a1* mRNA expression levels, while the S group displayed the opposite expression pattern. The WB analysis (Fig. S15) was performed to detect not only the cartilage-specific marker PRG4 and the osteogenic marker Collagen X, but also to further assess the expression of osteogenic proteins BMP2, distal-less homeobox 3 (DLX3), and Osterix (OSX). This comprehensive evaluation at the protein level allowed for an in-depth assessment of the ossification status of newly formed cartilage in each group. The results demonstrated that this therapeutic strategy significantly promoted cartilage regeneration while effectively inhibiting the ossification process, thereby confirming its dual therapeutic efficacy in cartilage repair.

Collectively, these multilevel analyses demonstrate that CSK@G treatment confers dual therapeutic advantages: promoting cartilage regeneration while simultaneously preventing further ossification of newly formed cartilage tissue. The CSK@G scaffold system establishes an optimal microenvironment that supports authentic cartilage formation and effectively inhibits progressive ossification.

## 4 Conclusions

This study employed additive manufacturing technology to construct a CSK@G biomimetic scaffold featuring continuous pore size gradients and spatial heterogeneity. Both *in vitro* and *in vivo* experiments demonstrated that this scaffold achieved region-specific tissue regeneration and directed stem cell differentiation through progressive porosity design. The mechanisms underlying scaffold-mediated osteochondral repair are primarily manifested in two aspects: first, the five-layer biomimetic structure precisely guides tissue regeneration in different regions while maintaining the cartilaginous phenotype; second, the incorporation of KGN-CS in the cartilage layer synergistically enhances stem cell recruitment and cartilage matrix formation. Compared to conventional homogeneous scaffolds, this gradient design overcomes the limitations of single-structure approaches and significantly improves repair efficacy [[60], [61], [62]]. A notable limitation is the absence of a CS@G control group in the current study. Although we have previously demonstrated that CS hydrogel alone exhibits limited chondrogenic capacity compared to KGN-loaded systems [24], those findings were obtained in conventional biomimetic osteochondral gradient scaffolds rather than cartilage microstructure-mimicking gradient systems. The cartilage microstructure-specific gradient architecture may introduce different interactions between CS and KGN that warrant independent validation. Future studies should incorporate the CS@G control within this cartilage microstructure-mimicking gradient system to confirm the synergistic effects. Additionally, the 12-week evaluation period, while demonstrating promising early-stage repair, may be insufficient to assess long-term scaffold integration, cartilage maturation, and biomechanical stability—critical factors for clinical translation that require extended observation periods (≥6 months). Future work will focus on addressing these limitations by incorporating additional control groups, extending evaluation timelines, further optimizing pore size gradient parameters, and integrating multifunctional bioactive factors to provide innovative clinical translation strategies for personalized osteochondral regenerative therapy.

## CRediT authorship contribution statement

**Hengyu Liu:** Investigation, Formal analysis, Data curation, Conceptualization. **Hongqing Qiao:** Methodology, Formal analysis, Data curation. **Rudong Li:** Software, Resources, Project administration. **Wenbo Yang:** Validation, Supervision. **Xingchen Guo:** Visualization, Validation, Supervision. **Yuhang Wang:** Software, Resources. **Nan Mei:** Validation, Supervision, Software. **Jincheng Wang:** Resources, Project administration, Methodology. **Fei Chang:** Writing – review & editing, Writing – original draft, Visualization, Validation, Funding acquisition.

## Ethics approval and consent to participate

All animal experimental protocols were approved by the Laboratory Animal Ethics Committee of the School of Basic Medical Sciences, Jilin University (No. 2024612) and conducted in accordance with the Guide for the Care and Use of Laboratory Animals (NIH Publication No. 8023, revised 1978).

## Consent for publication

All authors involved in this study have provided their consent for the publication of the research findings.

## Funding sources

This work was supported by the 10.13039/501100012166National Key Research and Development Program of China (No. 2022YFE0107700), the Scientific Research Project of the Jilin Provincial Department of Education (No. JJKH20262164BS).

## Declaration of competing interest

The authors declare that they have no known competing financial interests or personal relationships that could have appeared to influence the work reported in this paper.

## Data Availability

Data will be made available on request.
