# Supplementary material for: Kartogenin-loaded chitosan composite scaffold with cartilage-mimetic microstructure for layered osteochondral repair and cartilage phenotype maintenance
Source: Mater Today Bio. 2025 Dec 22;36:102727. doi: 10.1016/j.mtbio.2025.102727 (PMC12813363; doi:10.1016/j.mtbio.2025.102727)
Supplement: Multimedia component 1 [file mmc1.docx]

**Supplementary Materials**

**Kartogenin-loaded chitosan composite scaffold with cartilage-mimetic microstructure for layered osteochondral repair and cartilage phenotype maintenance**

Hengyu Liu^a,1^, Hongqing Qiao^a,1^, Rudong Li^b^, Wenbo Yang^a^, Xingchen Guo^a^, Yuhang Wang^a^, Nan Mei^c,d^, Jincheng Wang^a^, Fei Chang^a,*^

Hengyu Liu - hengyu24@mails.jlu.edu.cn

Hongqing Qiao - 1015414613@qq.com

Rudong Li - rdli24@mails.jlu.edu.cn

Wenbo Yang - [yangwbdr@163.com](mailto:yangwbdr@163.com)

Xingchen Guo - [guoxc24@mails.jlu.edu.can](mailto:guoxc24@mails.jlu.edu.can)

Yuhang Wang - wangyuhang25@mails.jlu.edu.cn

Nan Mei - N-mei@outlook.com

Jincheng Wang - [wangjinc@jlu.edu.cn](mailto:wangjinc@jlu.edu.cn)

^a^Department of Orthopedic Surgery, The Second Hospital of Jilin University, Changchun 130041, P.R. China

^b^Department of Gastrointestinal Nutrition and Hernia Surgery, The Second Hospital of Jilin University, Changchun 130041, P.R. China

^c^Department of Orthopaedic Surgery, Nara Medical University, Kashihara 634-8521, Japan

^d^Health Technology College, Jilin Sport University, Changchun 130022, P.R. China

^*^Corresponding authors at: Department of Orthopedic Surgery, The Second Hospital of Jilin University, Changchun 130041, P.R. China.
E-mail addresses： [changfei@jlu.edu.cn](mailto:changfei@jlu.edu.cn) (F. Chang).
^1^These authors (Hengyu Liu and Hongqing Qiao) contributed equally to this work.

**Supplementary Tables**

| Gene | Forward primer | Reverse primer |
| --- | --- | --- |
| *Gapdh* | 5’-GTATGATTCCACCCACGGCA-3’ | 5’-CCAGCATCACCCCACTTGAT-3’ |
| *Prg4* | 5’-TAACAGGGAAGATAGTGGCT-3’ | 5’-TTGATAAGAGGCTTTGATGG-3’ |
| *Col2a1* | 5’-AGCCTGGTGTCATGGGTTTC-3’ | 5’-GTCCCTTCTCACCAGCTTTGC-3’ |
| *Col10a1* | 5’-CTGTATAAGAATGGCACCCCTGTA-3’ | 5’-GCACTCCCTGAAGCCTGATC-3’ |
| *Ocn* | 5’-CAGAGCGACAGCATGAGGG-3’ | 5’-CCTCTGCCAGACTCTGCACC-3’ |
| *Bmp-2* | 5’-GCTTCCACCACGAAGAATCG-3’ | 5’-ATGGAAACCGCTGTCGTCTC-3’ |
| *Alp* | 5’-GCACTCCCACTTTGTCTGGA-3’ | 5’-TCCTGTTCAGCTCGTACTGC-3’ |

**Table. S1.** Primer sequences of genes

**Supplementary Figures**

**
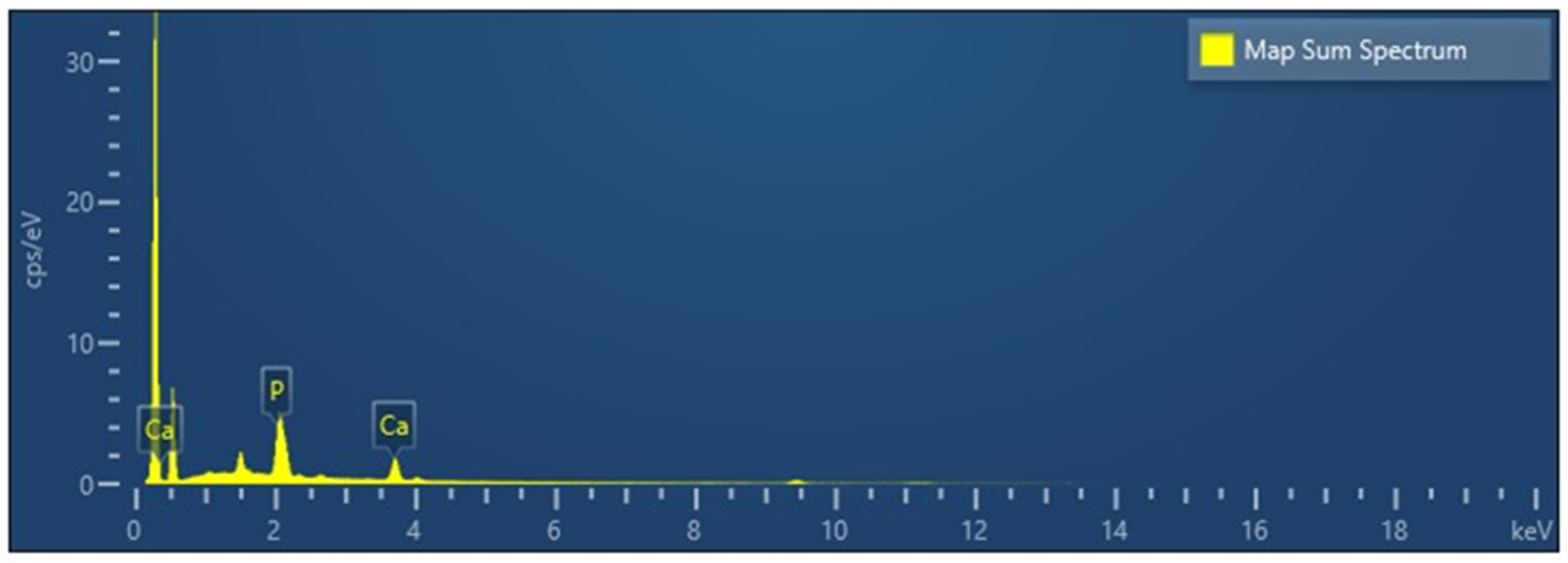
**

**Fig. S1.** EDS spectrum confirming the presence of calcium (Ca) and phosphorus (P) elements in the scaffold subchondral bone layer.


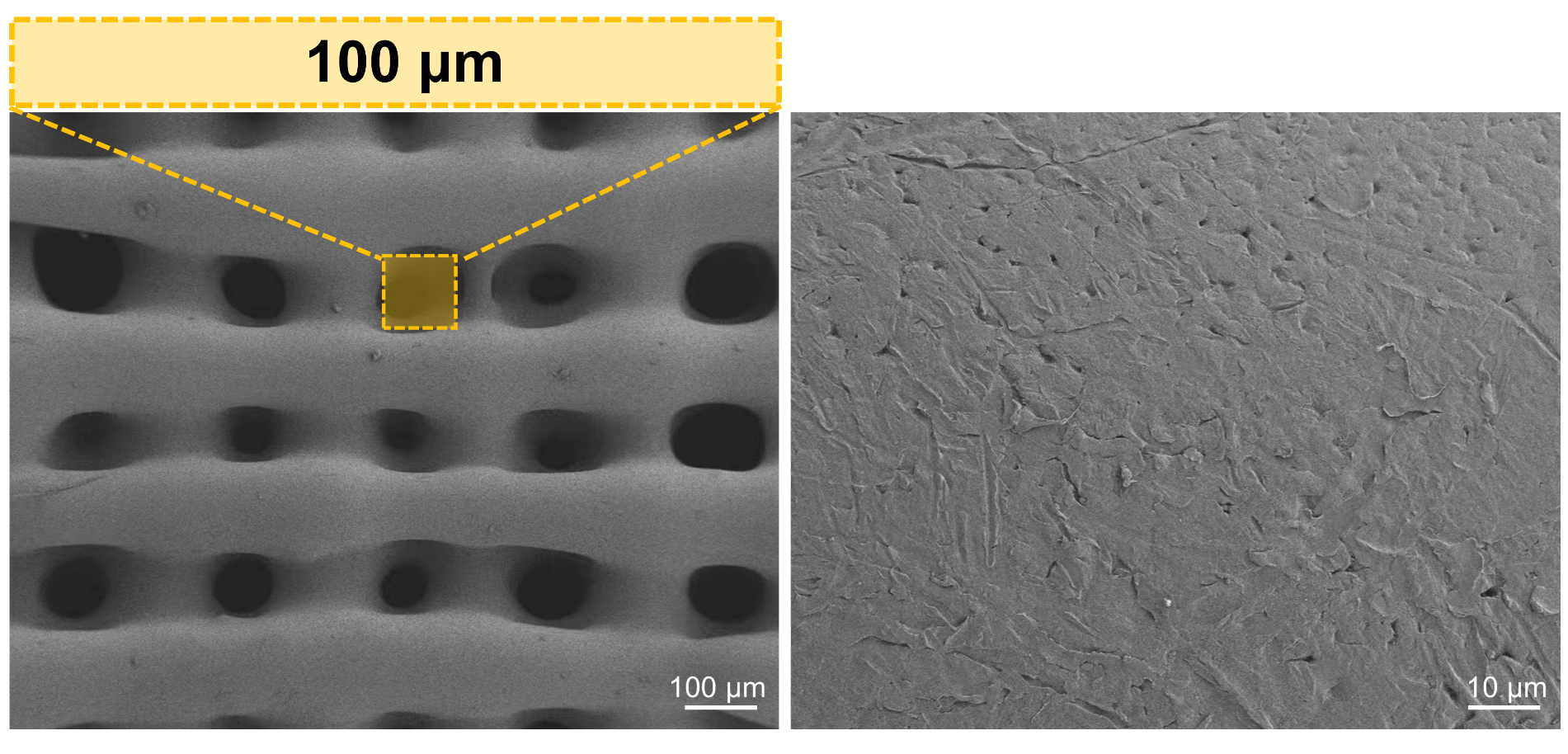


**Fig. S2.** SEM images of the PCL/HA layer with 100 μm pore size. The yellow boxes indicate the scaffold pore diameter.


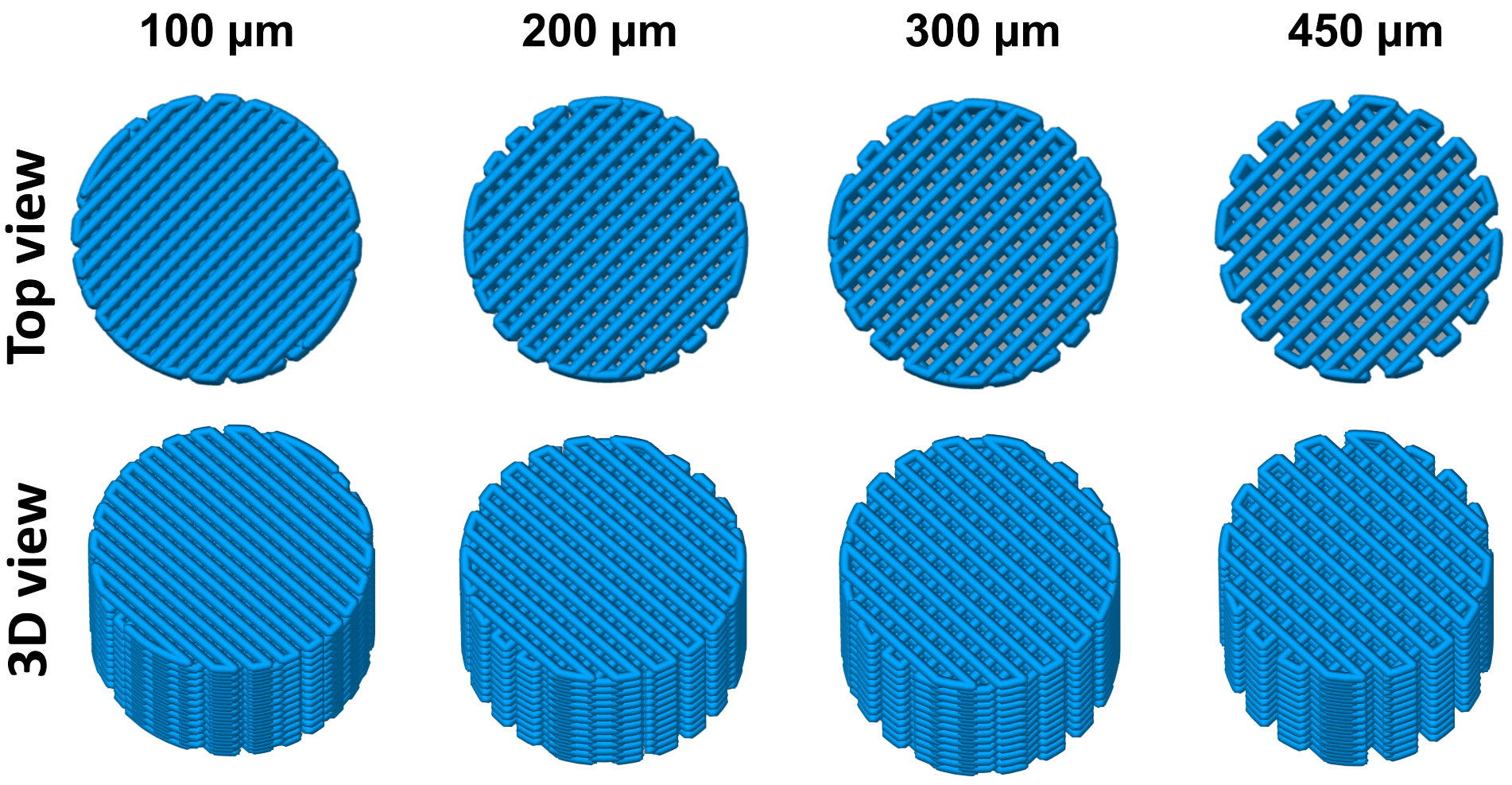


**Fig. S3.** Scaffold design with different pore sizes (100, 200, 300, and 450 μm) demonstrating pore distribution from top view and scaffold architecture from 3D perspective view.

**
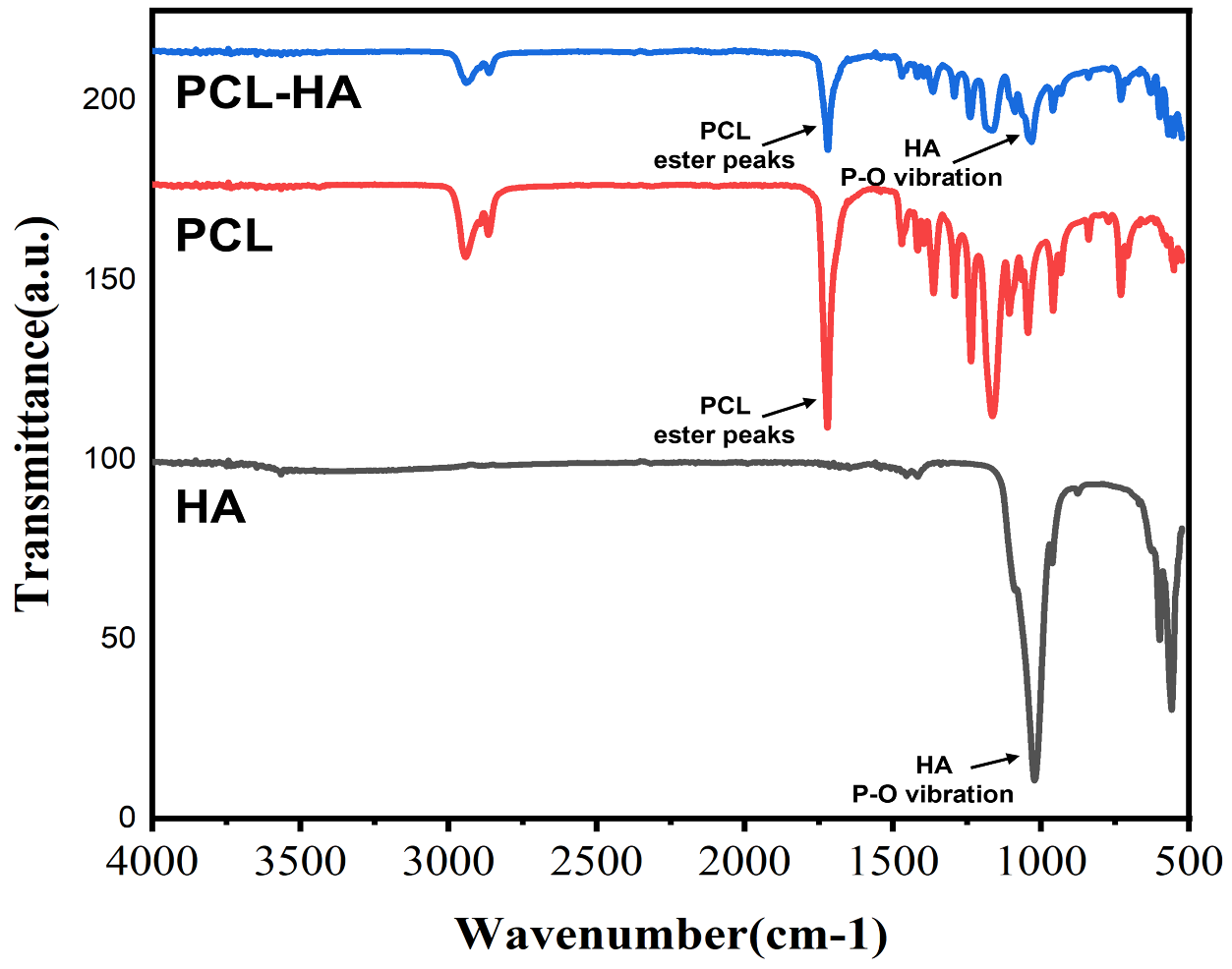
**

**Fig. S4.** FTIR spectra of HA, PCL, and PCL-HA composite materials. The spectra demonstrate successful incorporation of HA into PCL matrix, evidenced by the presence of characteristic PCL ester peaks (~1724 and ~2923 cm⁻¹) and HA phosphate vibration peaks (~1150 and ~960 cm⁻¹) in the PCL-HA composite spectrum.

**
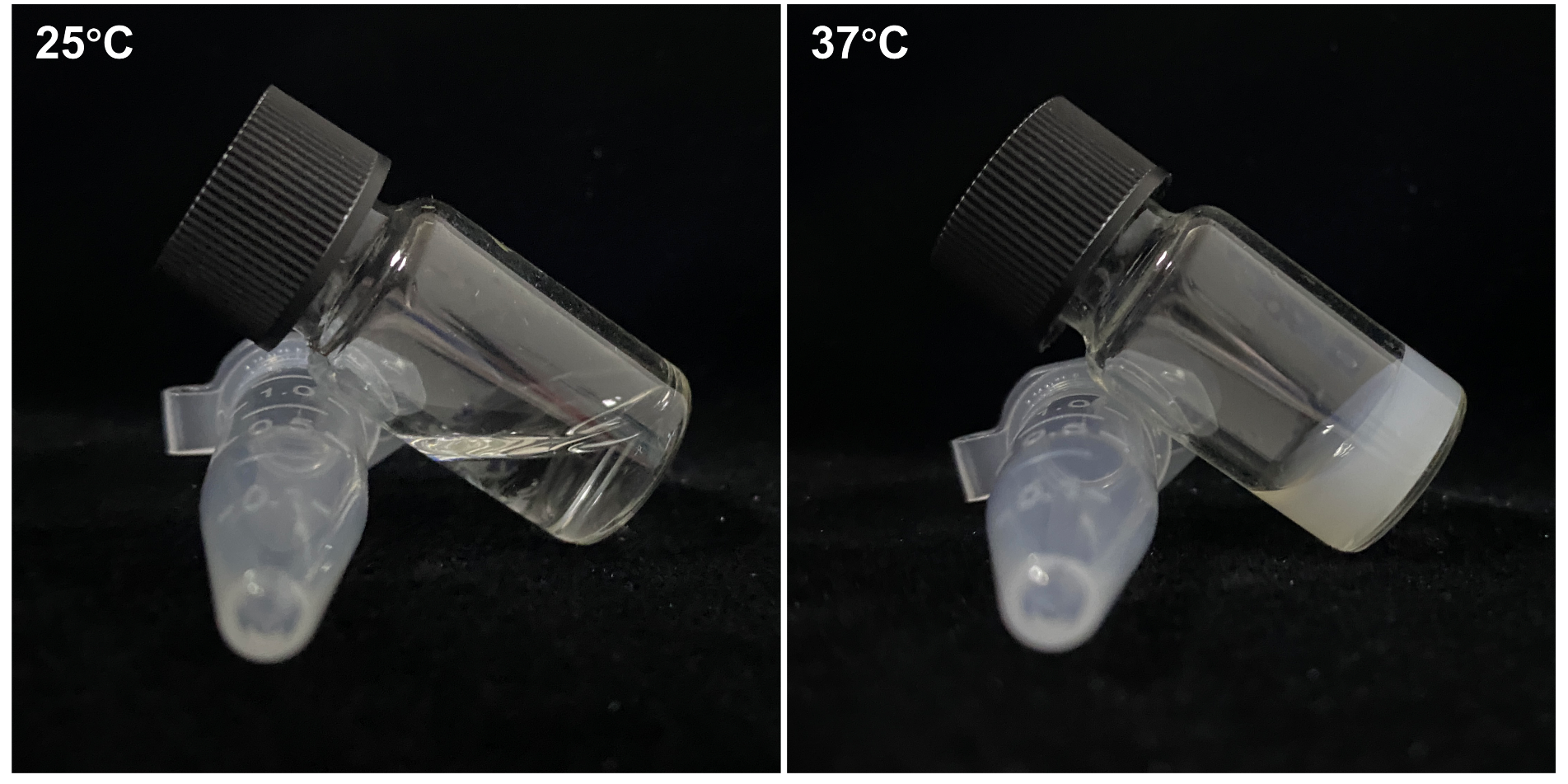
**

**Fig. S5.** Representative photographs showing the gross appearances of Kartogenin (KGN)-loaded chitosan (CS) hydrogels before and after gelation.


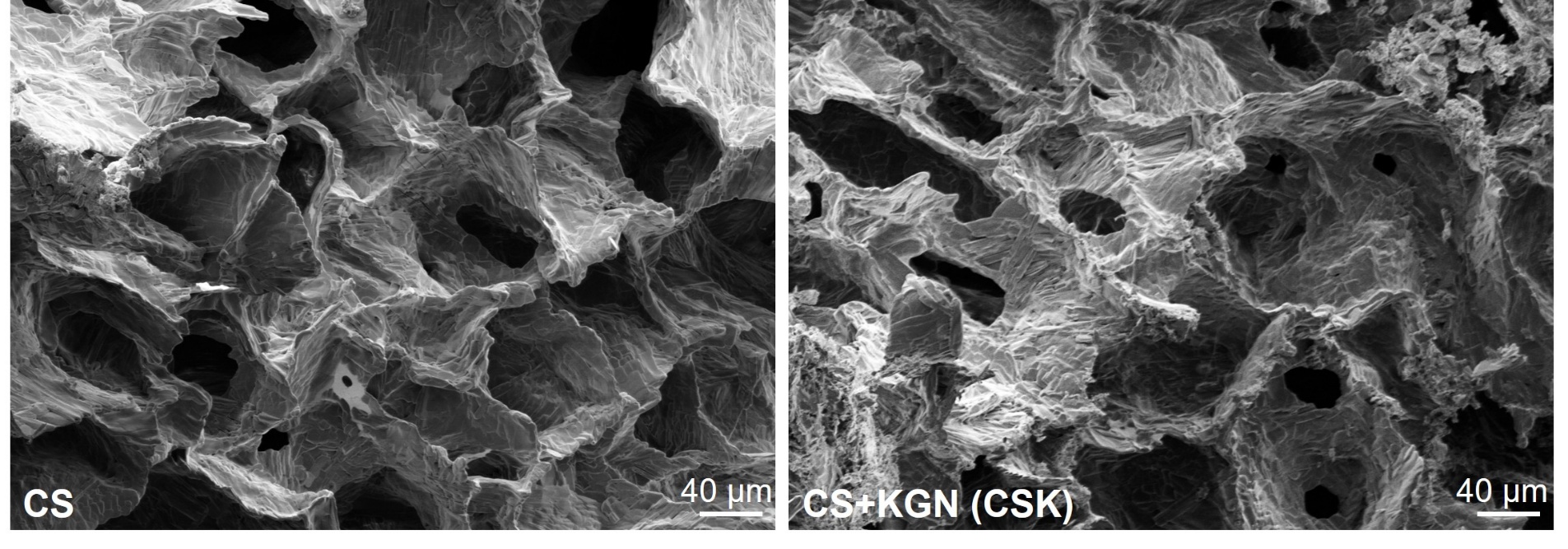


**Fig. S6.** SEM micrographs of chitosan hydrogels. (Left) Pure CS hydrogel showing the porous network structure, and (Right) KGN-loaded CS hydrogel (CSK) displaying similar morphological characteristics with maintained structural integrity after drug loading.

**
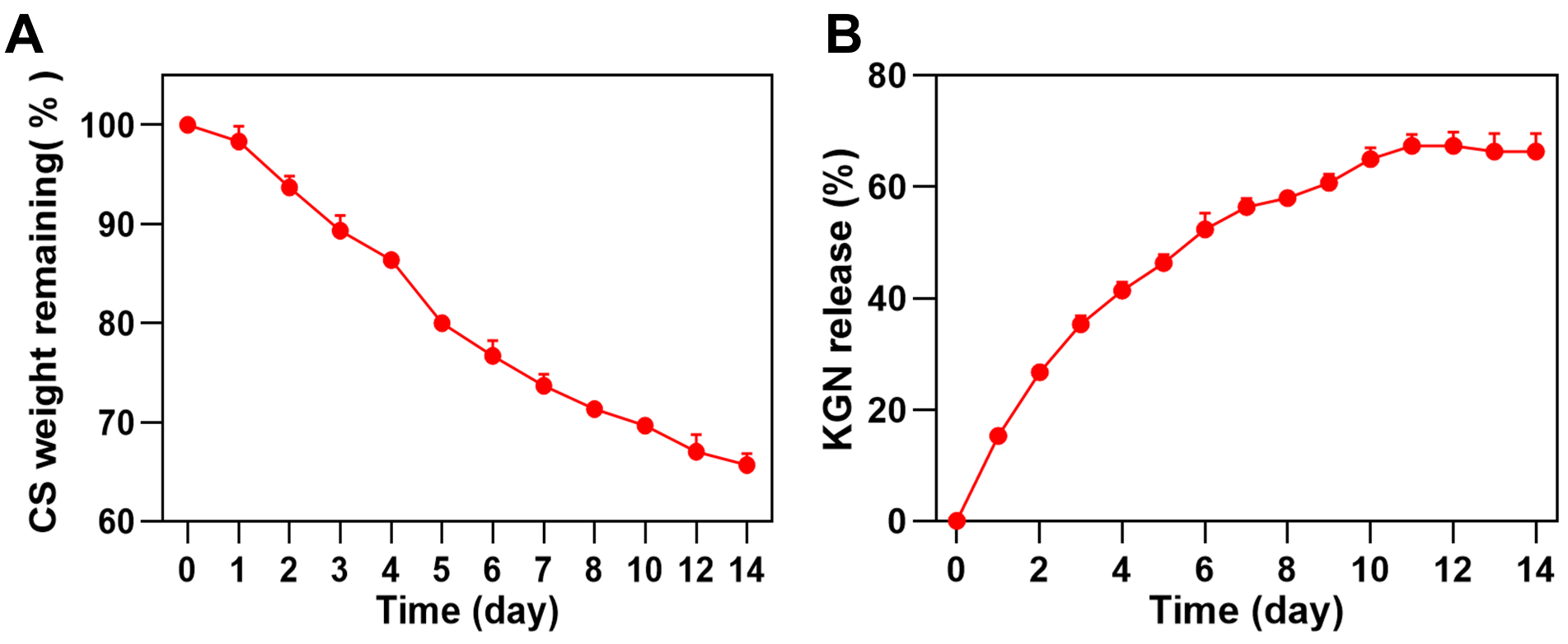
**

**Fig. S7.** Illustrates CS hydrogel degradation and KGN release kinetics over the experimental period, demonstrating controlled biodegradation and sustained drug delivery characteristics. (A) CS hydrogel weight remaining shows gradual degradation over time, indicating controlled biodegradation suitable for tissue engineering applications. (B) KGN release profile demonstrates sustained drug release, indicating controlled drug delivery suitable for cartilage regeneration applications.

**
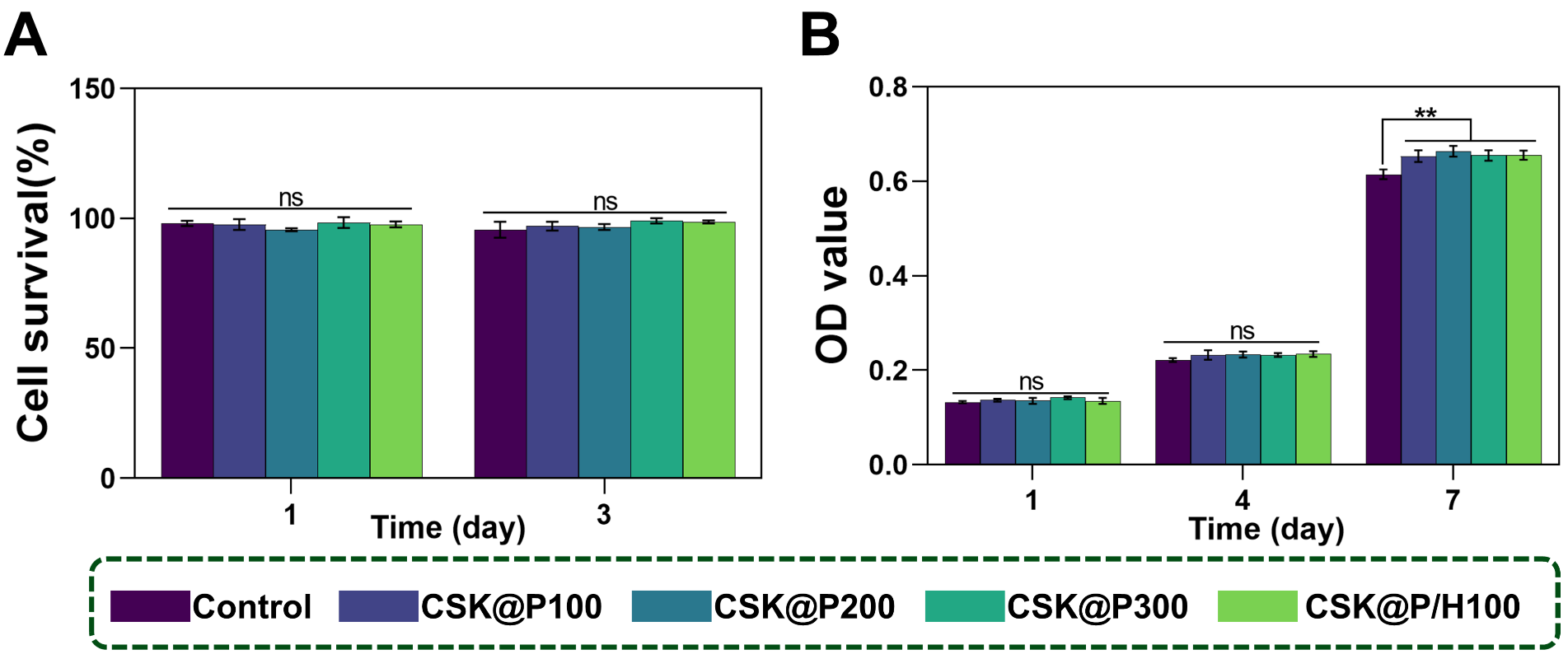
**

**Fig. S8.** Cell viability assessment of different scaffolds demonstrating excellent biocompatibility and KGN-mediated proliferation enhancement. (A) Cell survival rates show no significant differences between groups, indicating good biocompatibility of all scaffolds. (B) CCK-8 assay results demonstrate that KGN-loaded scaffolds significantly promote cell proliferation over time, with enhanced metabolic activity compared to control groups, particularly evident at later time points. (n=3, ***p* < 0.01, ns=not significant)


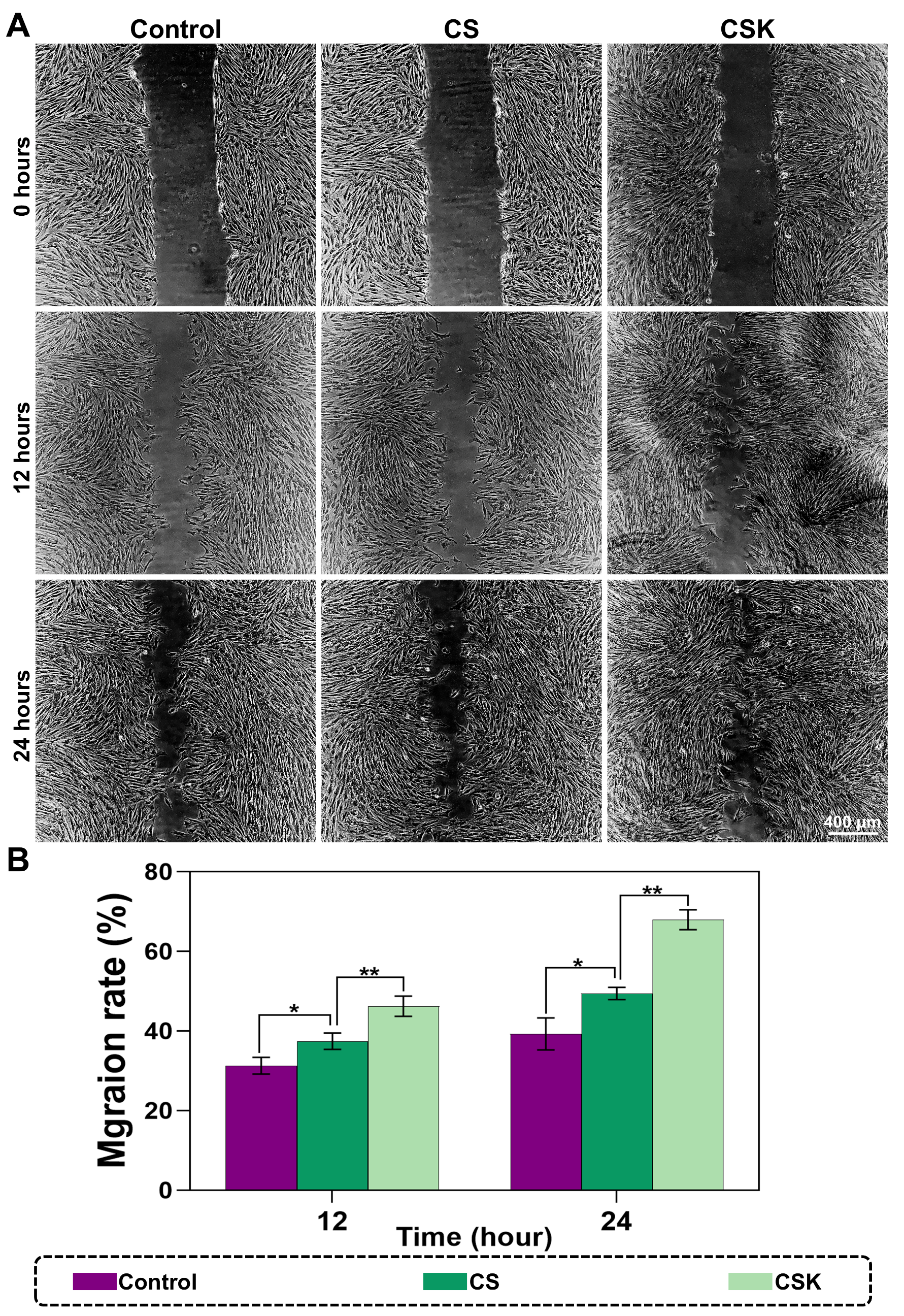


**Fig. S9.** Scratch wound healing assay and FITC-based cell exclusion zone migration assay. (A) Representative images showing scratch wound closure at 0, 12, and 24 hours across Control, CS, and CSK groups. (B) Quantitative migration rate analysis demonstrating time-dependent wound healing progression. (n=3, **p* < 0.05, ***p* < 0.01)


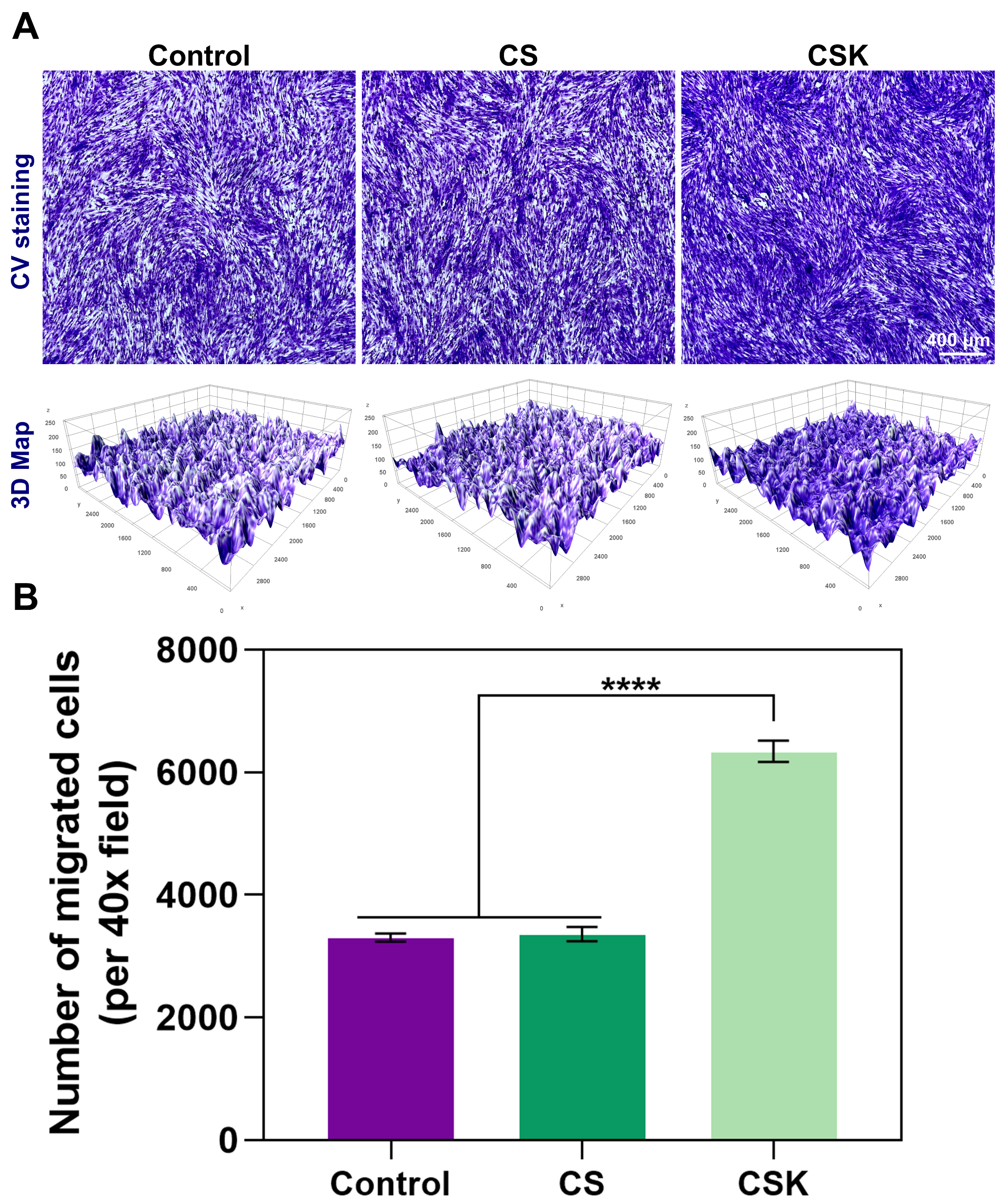


**Fig. S10.** Transwell migration assay of different treatment groups. (A) Crystal violet (CV) staining of migrated cells (Control, CS, CSK groups). Upper: microscopic images (40×); Lower: 3D mapping visualization. (B) Quantitative analysis of migrated cell numbers per field. (*****p* < 0.0001)

**
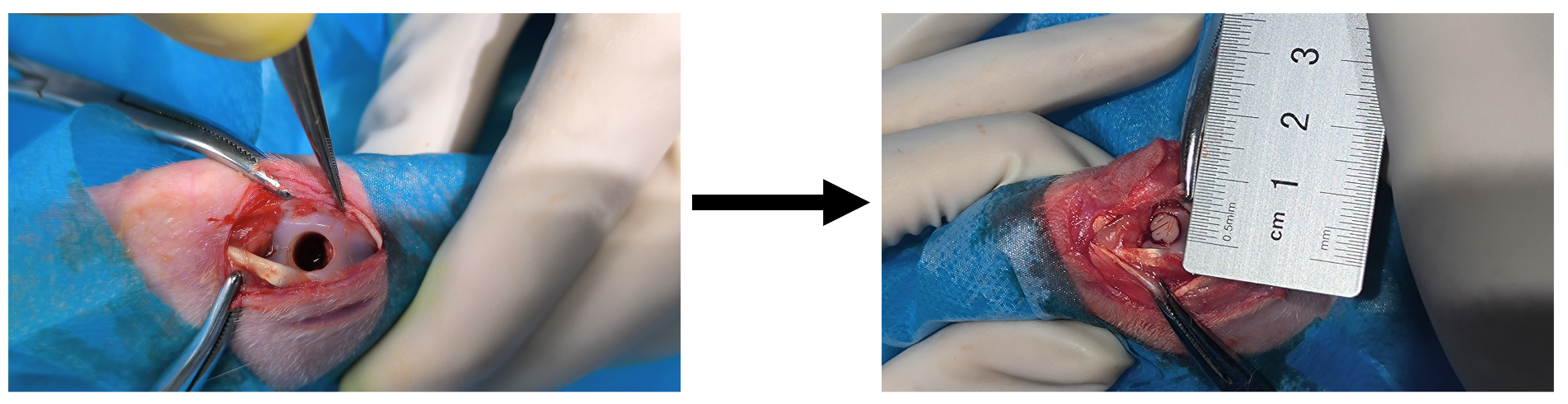
**

**Fig. S11.** Intraoperative macroscopic images showing osteochondral defect creation and scaffold implantation.


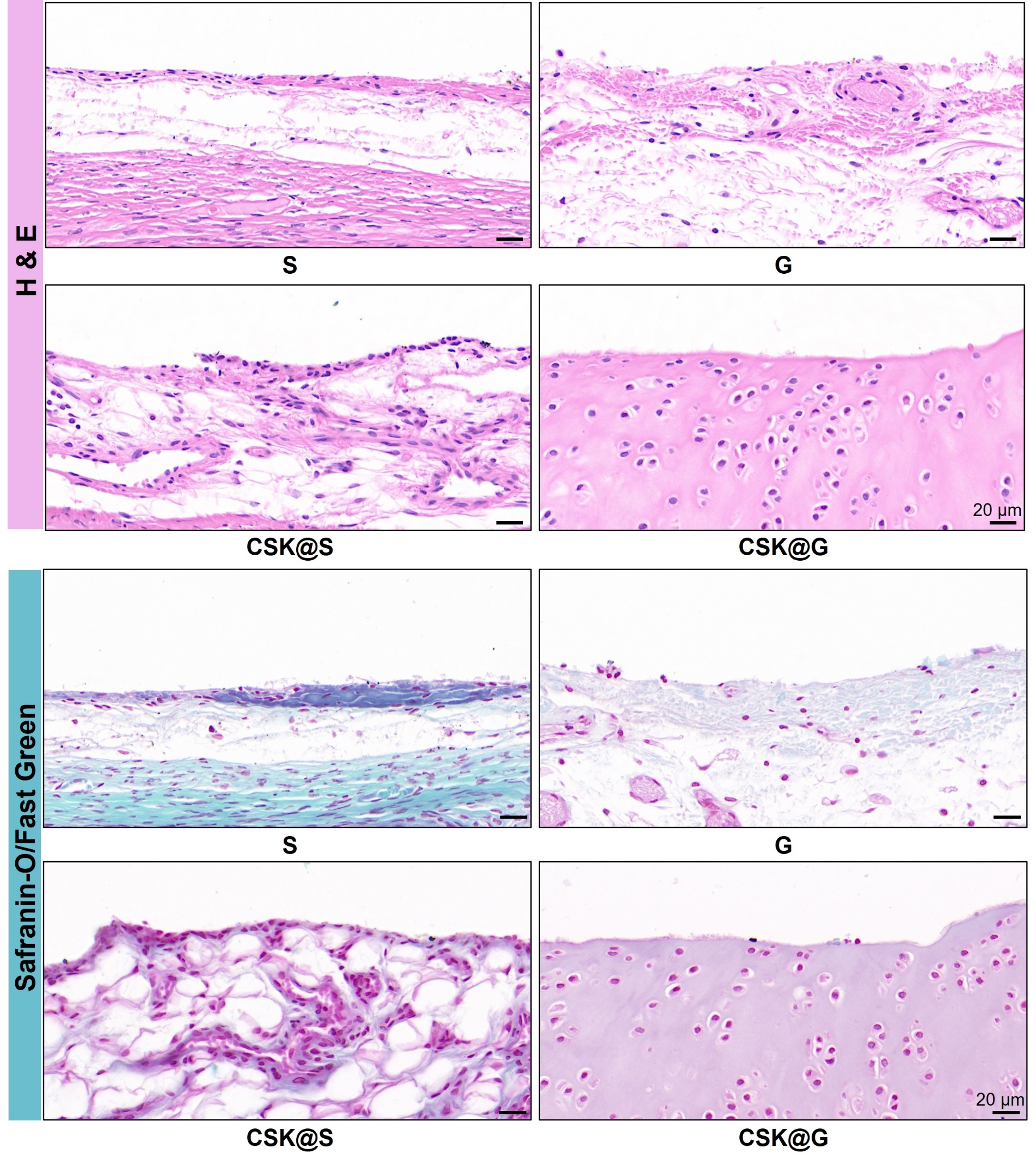
**Fig. S12.** Representative high-magnification HE and Safranin O/Fast Green staining images of regenerated cartilage in each group at 12 weeks post-surgery.


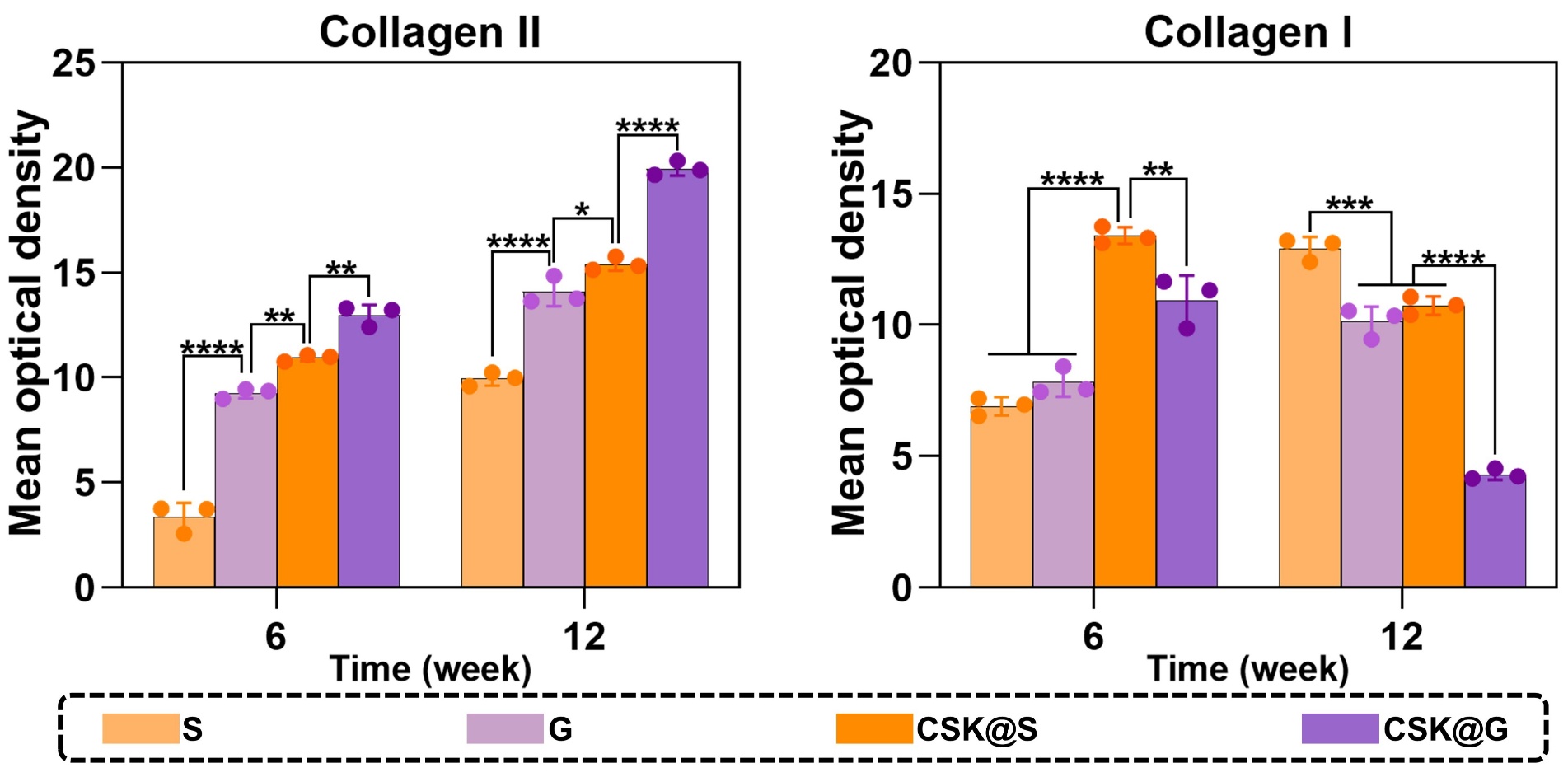
**Fig. S13.** Quantitative analysis of mean optical density (MOD) for immunohistochemical staining of Collagen II and Collagen I in the cartilage repair region of each experimental group at 6 and 12 weeks post-implantation. (n=3, **p* < 0.05, ***p* < 0.01, ****p* < 0.001, *****p* < 0.0001)

**
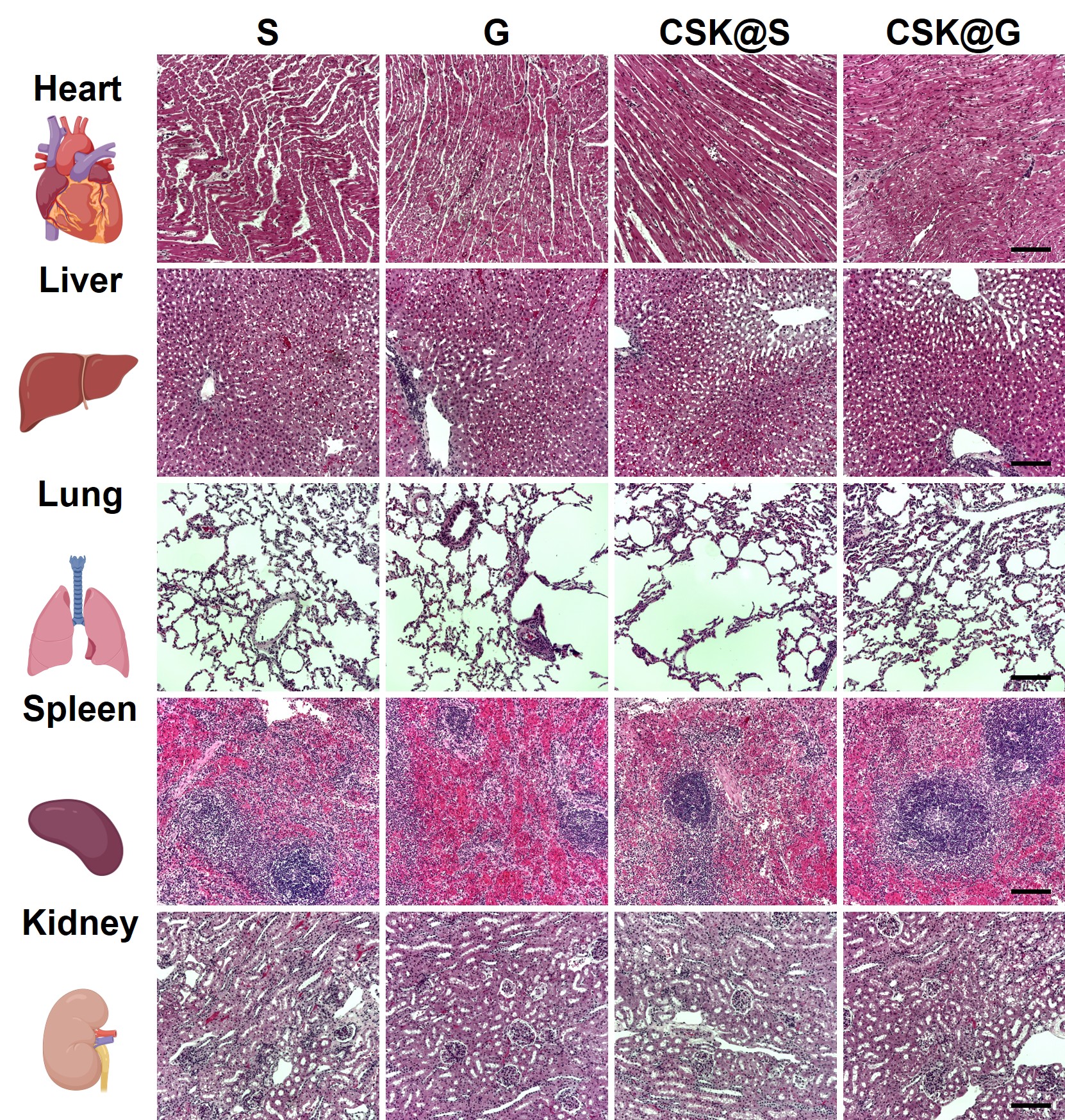
**

**Fig. S14.** Histological examination (HE staining) of major organs (heart, liver, lung, kidney, and spleen) from New Zealand white rabbits in each experimental group at 12 weeks post-implantation. (n=3, scale bar: 100 μm)


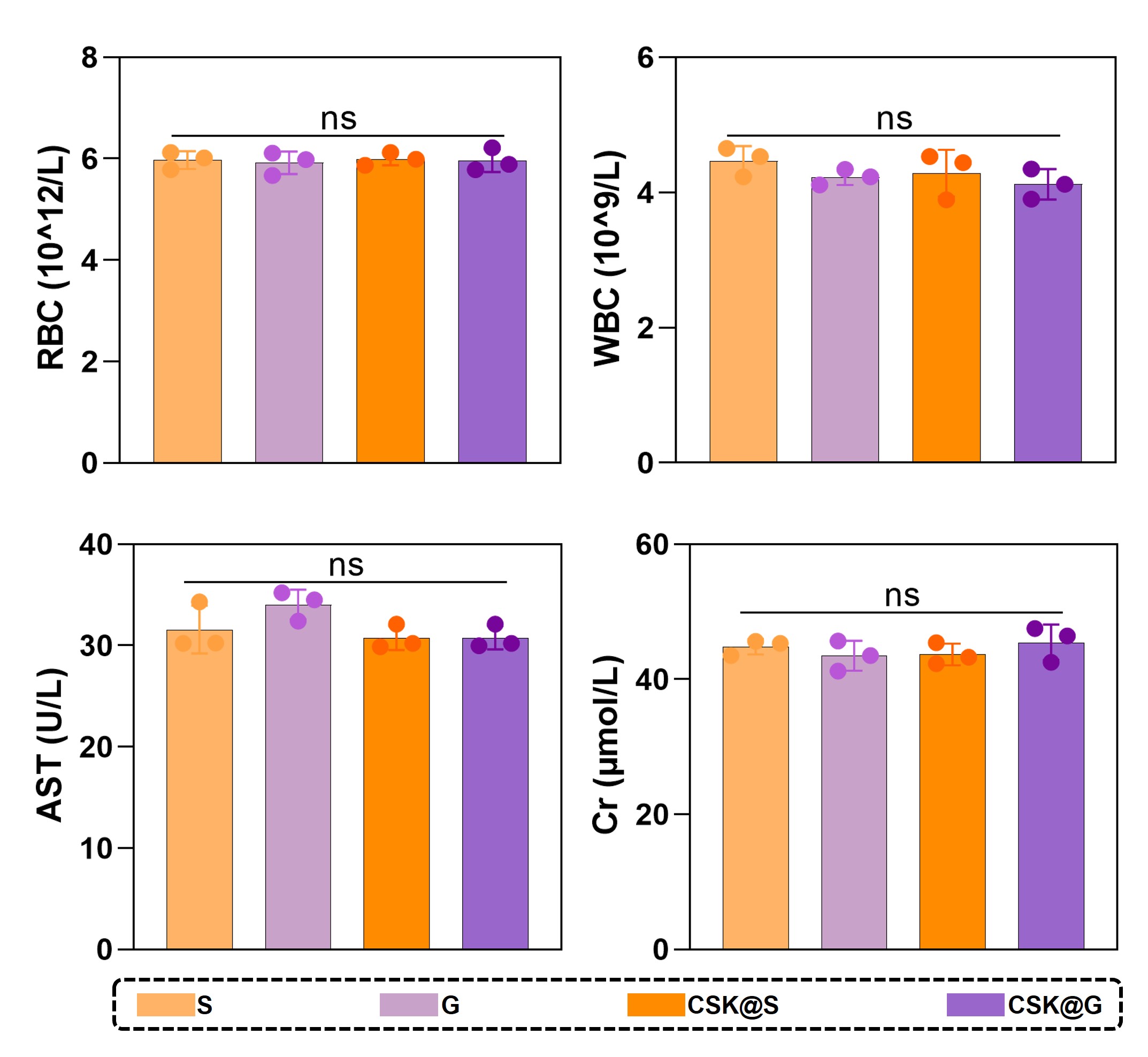


**Fig. S15.** Hematological and biochemical analyses confirmed systemic safety with red blood cells (RBC), white blood cells (WBC), and hepatorenal function indicators (AST, Cr) remaining within normal physiological ranges at 12 weeks post-implantation. Data demonstrate excellent biocompatibility with negligible systemic toxicity throughout the healing period. (n=3) (ns, not significant)


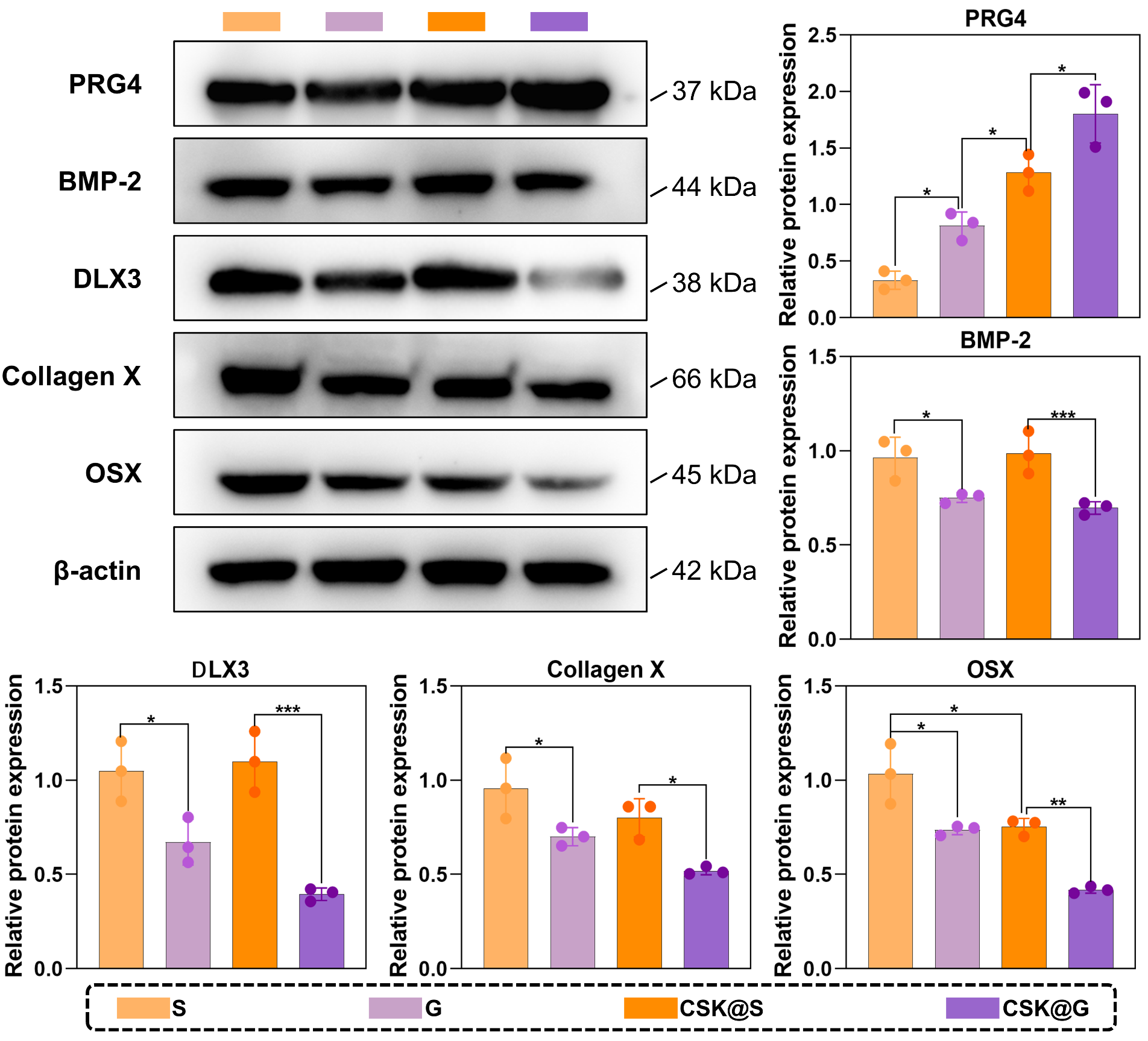


**Fig. S16.** Western blot analysis of chondrogenesis and cartilage ossification markers in newly formed cartilage tissue at 12 weeks post-surgery. Protein expression levels of PRG4, BMP-2, DLX3, Collagen X, and OSX were detected and quantified, with β-actin serving as the loading control. (n=3, **p* < 0.05, ***p* < 0.01, ****p* < 0.001)
